# CARD9-dependent macrophage plasticity regulates effective fungal clearance

**DOI:** 10.1172/JCI188827

**Published:** 2025-12-02

**Authors:** Lu Zhang, Zhichun Tang, Yi Zhang, Wenjie Liu, Haitao Jiang, Li Yu, Kexin Lei, Yubo Ma, Yang-Xin Fu, Ruoyu Li, Wenyan Wang, Fan Bai, Xiaowen Wang

**Affiliations:** 1 Department of Dermatology and Venerology, Peking University First Hospital, Beijing, China.; 2 Research Center for Medical Mycology, Peking University, Beijing, China.; 3 Beijing Key Laboratory of Molecular Diagnosis on Dermatoses, Beijing, China.; 4 National Clinical Research Center for Skin and Immune Diseases, Beijing, China.; 5 Biomedical Pioneering Innovation Center (BIOPIC) and School of Life Sciences, Peking University, Beijing, China.; 6 School of Basic Medical Sciences and; 7 State Key Laboratory of Molecular Oncology, Tsinghua University, Beijing, China.; 8 Peking-Tsinghua Center for Life Sciences (CLS), Peking University, Beijing, China.; 9 State Key Laboratory of Metabolic Dysregulation and Prevention and Treatment of Esophageal Cancer, BIOPIC, Peking University, Beijing, China.; 10 Peking University Beijing-Tianjin-Hebei Biomedical Pioneering Innovation Center, Tianjin, China.

**Keywords:** Dermatology, Immunology, Infectious disease, Fungal infections, Macrophages, Skin

## Abstract

The role of CARD9 in the pathogenesis of various chronic fungal infections has been established; however, the precise mechanisms underlying the pathobiology of these infections remain unclear. We investigated the specific cellular mechanisms by which CARD9 deficiency contributes to the pathogenesis of chronic fungal infections. Using single-cell RNA-seq, we analyzed the immune cell profiles in skin lesions from both murine and human samples. We focused on macrophage differentiation and signaling pathways influenced by CARD9 deficiency. We found that CARD9 deficiency promoted the differentiation of high levels of triggering receptor expressed on myeloid cells 2 (TREM2^hi^) monocyte–derived macrophages after fungal stimulation, impairing their antifungal functions and inducing exhaustion-like Th1 cells. Mechanistically, NF-κB pathway activation was restricted in CARD9-deficient macrophages, leading to enhanced CREB activation, which, in turn, exerted a positive regulatory effect on *Trem2* expression by activating C/EBPβ. Notably, targeting TREM2 enhanced the antifungal immune response in vivo and in vitro, thereby alleviating the severity of CARD9-deficient subcutaneous dematiaceous fungal infection. Our findings highlight the important role of CARD9 in regulating cutaneous antifungal immunity and identify potential targets for immunotherapy in chronic dematiaceous fungal infections.

## Abstract 2

This study unveils a novel mechanism by which CARD9 regulates macrophage phenotype and antifungal immunity, highlighting their potential as therapeutic targets.

## Graphical abstract

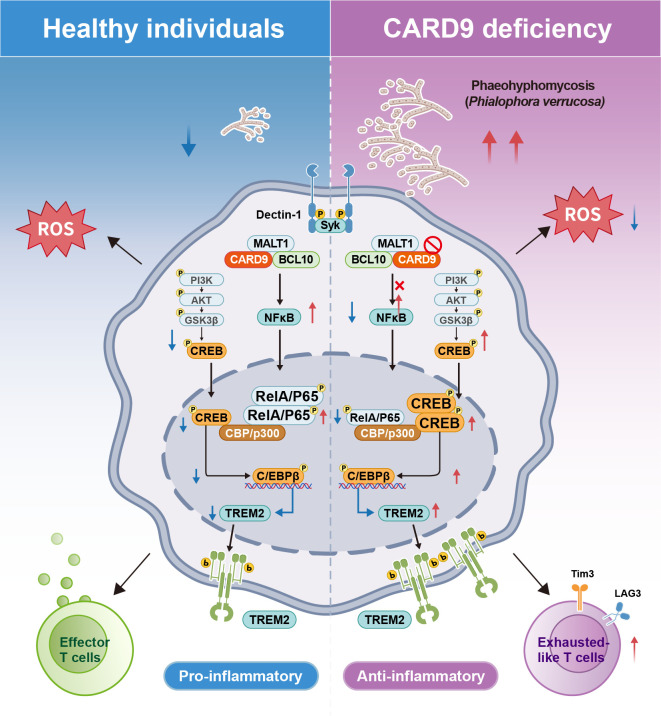

## Introduction

Fungal infections are a crucial and growing global health concern because of the various pathogens and clinical manifestations (1–3). Recent estimates indicate more than 6.55 million individuals worldwide face life-threatening fungal infections annually (4), underscoring the magnitude of this issue. With advances in genetics and immunology, the critical role of genetic susceptibility in patients has been uncovered, particularly in patients with deficiencies in caspase recruitment domain-containing protein 9 (CARD9), which are associated with severe intractable fungal infections and high mortality rates (1, 5, 6). CARD9 is a crucial signaling adaptor that functions downstream of several C-type lectin receptors and plays a vital role in host immune responses against fungal pathogens (5, 7, 8). However, the comprehensive mechanisms by which CARD9 influences antifungal immunity have not been fully elucidated.

Macrophages, the primary cell type expressing CARD9, play a crucial role in antifungal immunity via direct and indirect mechanisms (9–11). Previous studies have shown that although loss of CARD9 markedly impairs the fungicidal capacity of macrophages (12–16), it does not notably affect their recruitment or phagocytic functions (17). However, more recent research using the candidiasis model reported that CARD9 deficiency led to defective monocyte aggregation at day 1 after infection, followed by abnormal accumulation of Ly6C^+^ monocytes and MHCII^+^Ly6C^+^ monocyte–derived cells by day 4 in the infected brain (18). These findings highlight inconsistencies in the reported effects of CARD9 deficiency on macrophage-mediated antifungal responses across different infection models. Triggering receptor expressed on myeloid cells 2 (TREM2), a myeloid cell surface receptor, has been identified as an important immune signaling hub in several pathological conditions (19, 20). Its effect on macrophage function remains a topic of considerable debate, with evidence suggesting TREM2 exerts opposing effects in different disease states. Some studies have suggested TREM2 negatively regulates TLR signaling, thereby suppressing proinflammatory mediator secretions and anti-infective functions (20–25). Conversely, recent studies have indicated TREM2 can induce bacterial phagocytosis, which is crucial for pathogen clearance and inflammation onset (26, 27). However, the influence of TREM2 on host antifungal immune function remains unexplored and warrants further investigation.

Host immune responses to fungal pathogens involve a complex interplay between innate and adaptive immunity. Adaptive immunity, particularly Th1-related and Th17-related cellular responses, is crucial for robust antifungal capabilities (28–30). CARD9 serves as a critical bridge between innate and adaptive immunity. Previous research has suggested that CARD9 deficiency impairs Th1 and Th17 cell differentiation and compromises essential secretion of cytokines such as IFN-γ, IL-17A, and IL-22, thereby weakening the adaptive antifungal immune response in the host (6, 15, 31, 32). Nevertheless, some patients with CARD9 deficiency have normal Th17 cell differentiation (33–38). These inconsistencies highlight the need for further investigations into how CARD9 deficiency affects adaptive antifungal immunity.

Given the high prevalence and treatment resistance of dematiaceous fungal infections in patients with CARD9 deficiency at our center, we used single-cell RNA-seq (scRNA-seq) to investigate the local immune landscape in the skin lesions of both murine models and human patients with this infection. This study revealed the function of CARD9 in regulating the differentiation of macrophages by modulating the balance between the NF-κB/P65 and CREB-C/EBPβ pathways. Consequently, TREM2^hi^ macrophages are enriched in individuals with CARD9 deficiency, which affects innate and adaptive antifungal immune responses. Moreover, the administration of TREM2 agonists can delay the progression of CARD9-deficient dematiaceous fungal infections. Our findings thus reveal the regulatory mechanisms underlying TREM2 expression and its influence on antifungal immune responses, identifying a potential target for immunotherapy in patients with chronic CARD9-related dematiaceous fungal infections.

## Results

### CARD9 is necessary for defense against subcutaneous dematiaceous fungal infection.

**Figure 1:**
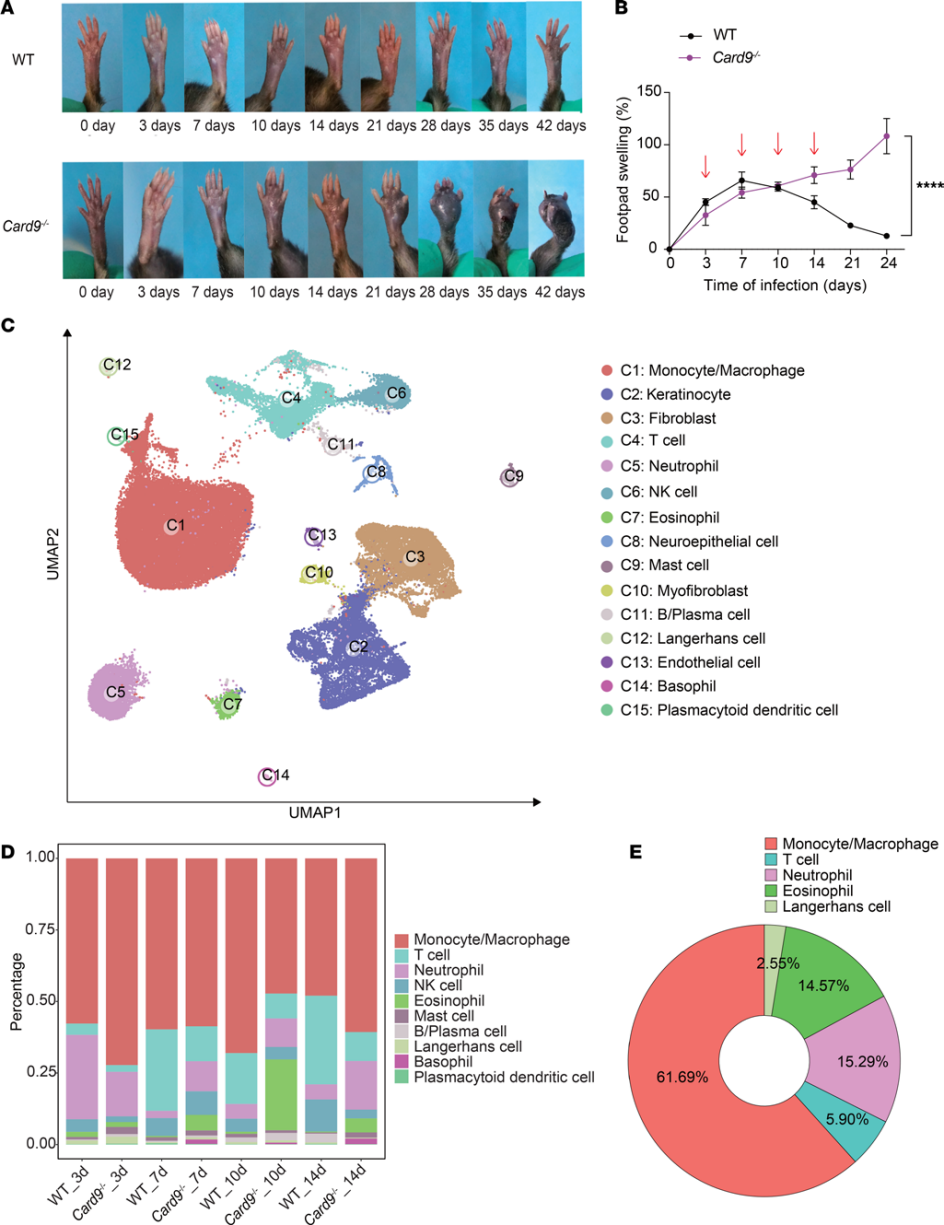
CARD9 is necessary for defense against subcutaneous dematiaceous fungal infection. (**A**) Natural course of subcutaneous infection with *P*. *verrucosa* in WT and *Card9*^–/–^ mice. (**B**) Footpad swelling of *P*. *verrucosa*–infected WT and *Card9*^–/–^ mice at different time points after infection (*n* = 3). (**C**) The uniform manifold approximation and projection (UMAP) plot presents the projection of 59,396 high-quality cells from 8 scRNA-seq samples, comprising 4 samples each from WT and *Card9*^–/–^ group. Each point on the plot represents a single cell, with colors varying according to distinct cell types. (**D**) The stacked bar chart shows the percentage distribution of 10 immune cell types across all samples. The colors representing each cell type are consistent with those shown in **C**. (**E**) The pie chart depicts the distribution of Card9^+^ cells among immune cell subsets within lesional skin. Data were integrated from all samples. Data are representative of 3 independent experiments and are shown as the mean ± SD. ****P < 0.0001, by 2-way ANOVA (**B**).

Previous studies indicated that patients with CARD9 deficiency are susceptible to severe dematiaceous fungal infections; however, its underlying mechanisms remain poorly understood. *Phialophora verrucosa* is the most commonly identified causative fungus in patients with CARD9 deficiency with phaeohyphomycosis (32). To investigate the role of CARD9 in shaping host protective immunity against dematiaceous fungi, we first modeled subcutaneous phaeohyphomycosis with footpad inoculation of *P*. *verrucosa* in WT and *Card9*-knockout (*Card9*^–/–^) mice. After fungal inoculation, the 2 groups exhibited different patterns of footpad swelling. WT mice showed more pronounced swelling on day 3, with comparable levels between groups on day 7, followed by gradual resolution. However, *Card9*^–/–^ mice experienced progressive deterioration, with increased swelling on day 10 and more pronounced swelling on day 14 and thereafter (Figure 1, A and B).

To elucidate the cellular and molecular mechanisms underlying CARD9-mediated antifungal immune responses during subcutaneous *P*. *verrucosa* infection, we conducted scRNA-seq of total cells in mouse footpads at 3, 7, 10, and 14 days after infection (Figure 1B). After data preprocessing and quality control, we partitioned the cells into 15 major clusters and labeled them based on representative marker genes, including 10 immune cell clusters and 5 nonimmune cells (Figure 1C). Overall, on day 3 after infection, neutrophils, monocytes, and macrophages were the predominant immune cells in both mouse strains, with *Card9*^–/–^ mice having fewer neutrophils and more monocytes and macrophages than did WT control mice (Figure 1D). On day 7 after infection, the proportions of T and NK cells increased in the skin lesions of WT mice and remained elevated through days 10 and 14 (Figure 1D). In contrast, *Card9*^–/–^ mice had reduced T and NK cell infiltration but an increased proportion of eosinophils in the lesions compared with WT mice (Figure 1D).

To elucidate the role of CARD9 in modulating the recruitment and function of local immune cells in lesions, we analyzed the distribution of Card9-expressing cells in this model. Macrophages constituted the predominant population, comprising 61.69% of Card9^+^ cells (Figure 1E). This predominant expression underscores macrophages as the pivotal cellular subset for subsequent in-depth analysis.

### TREM2^hi^ macrophages display antiinflammatory signatures and are increased in Card9^–/–^ mice.

**Figure 2:**
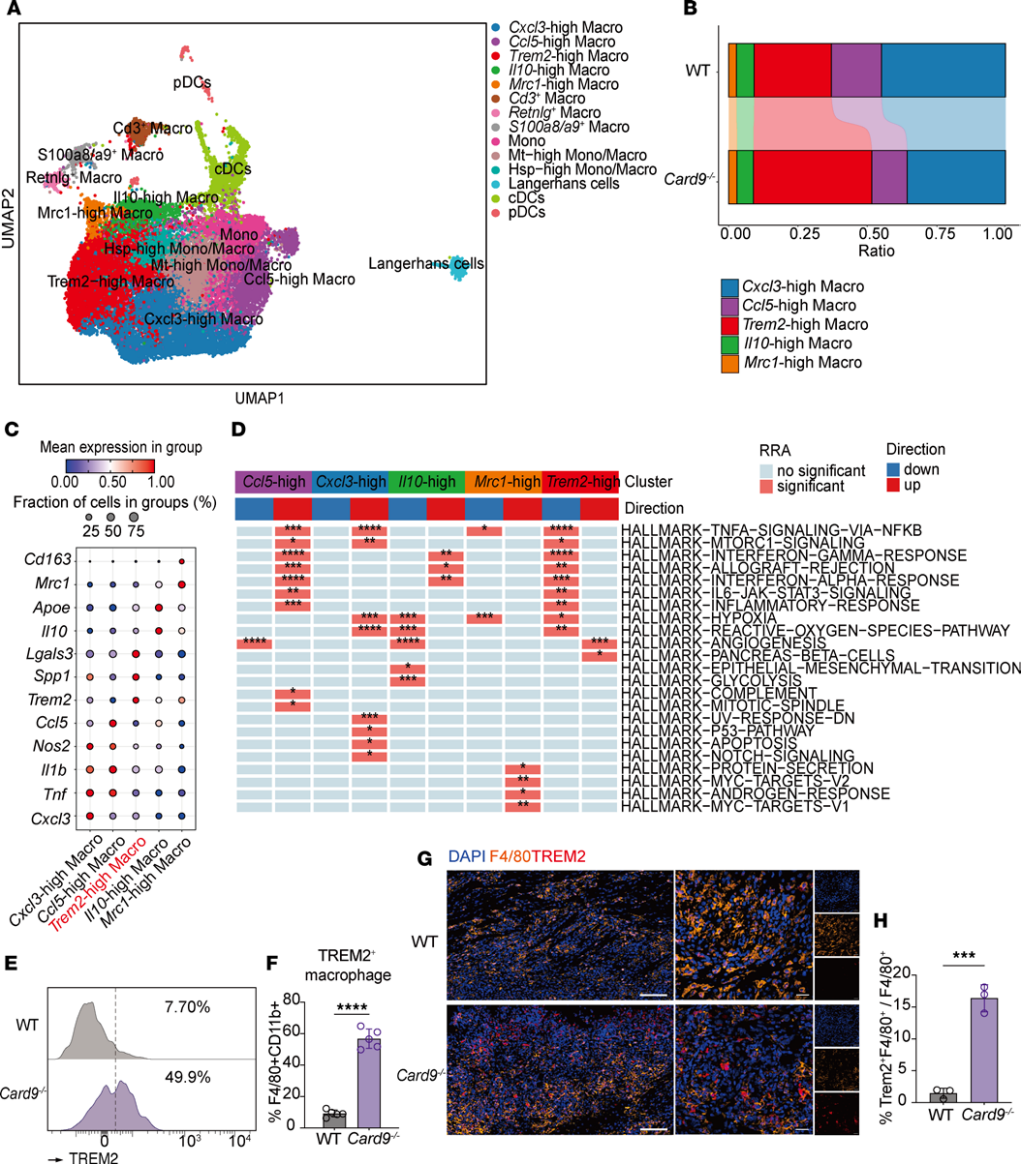
TREM2^hi^ macrophages display antiinflammatory signatures and are increased in *Card9*^–/–^ mice. (**A**) UMAP of myeloid cells. Each dot represents a single cell, with colors varying according to distinct cell subpopulations. (**B**) The Sankey diagram illustrates the proportional differences between the major macrophage subpopulations across the 2 groups. (**C**) The bubble chart shows the mean relative expression of signature genes across the major macrophage subpopulations. (**D**) The heatmap illustrates the differences in HALLMARK gene set scores among the major macrophage subpopulations, with scores calculated via the irGSEA R package (https://github.com/chuiqin/irGSEA/). Only gene set scores exhibiting differences across subpopulations are presented. (**E** and **F**) Representative flow cytometry histogram plots for TREM2 staining (**E**) and frequency of TREM2^+^ macrophage subsets (**F**) in murine footpad lesions at day 10 after infection. One data point denotes a result from 1 mouse (*n* = 5). RRA, robust rank aggregation. (**G** and **H**) Staining of TREM2^+^ macrophages (TREM2 and F4/80) in murine lesions. Scale bars: 100 μm (left) and 20 μm (right). Bar plots show the quantification results (**H**). One data point represents the statistical result of 1 field of view (*n* = 3 fields analyzed per condition). Data are shown as the mean ± SD. ***P < 0.001 and ***P < 0.0001, by 2-tailed Student’s *t* test (**F** and **H**).

Macrophages, a pivotal cell type in antifungal immunity, are among the main expressers of CARD9. We conducted further subpopulation analyses and identified 5 distinct macrophage subsets: *Cxcl3*^hi^, *Ccl5*^hi^, *Trem2*^hi^, *Il10*^hi^, and *Mrc1*^hi^ macrophages (Figure 2A). The proportions of *Cxcl3*^hi^ macrophage and *Ccl5*^hi^ macrophage were higher in WT mice, whereas the TREM2^hi^ macrophage subset was considerably more abundant in *Card9*^–/–^ murine lesions (Figure 2B). Notably, the TREM2^hi^ macrophage subset was characterized by high expression of *Trem2*, *Lgals3*, and *Spp1* (Figure 2C), a transcriptional profile consistent with the gene signature previously described for skin monocyte–derived macrophages (39). In addition, the analysis of monocyte-associated gene programs across macrophage subsets provided further indications that this population represents a monocyte-derived macrophage lineage (Supplemental Figure 1A; supplemental material available online with this article; https://doi.org/10.1172/JCI188827DS1).

HALLMARK gene set scoring among the major macrophage subsets revealed that the TREM2^hi^ macrophages exhibited notably reduced activities in the NF-κB signaling pathway and pathways related to proinflammatory cytokines, such as TNF-α, IFN-γ, and IL-6 (Figure 2D). Further flow cytometry analysis demonstrated that the proportion of TREM2^+^ macrophages among all macrophages was markedly higher in *Card9*^–/–^ murine lesions than in WT murine lesions on day 10 after infection (Figures 2, E and F), whereas no differences were observed prior to infection (Supplemental Figure 1B). Multiplex immunofluorescence (mIHC) experiments also showed higher TREM2 expression and its colocalization with the macrophage marker F4/80 in lesions of *Card9*^–/–^ mice (Figure 2, G and H).

The collective findings indicate CARD9 deficiency does not notably affect macrophage recruitment to the infection site but rather substantially alters macrophage phenotypes in response to subcutaneous *P*. *verrucosa* infection. A similar pattern of increased recruitment but impaired functional responses has been reported in CARD9-deficient mice challenged with *C*. *albicans* (18). Together, these observations suggest CARD9 regulates macrophage plasticity, potentially impairing antifungal effector functions.

Neutrophils are another type of immune cell that mainly expresses CARD9 and plays a critical role in antifungal immunity. We performed a detailed subpopulation analysis of neutrophils to investigate their heterogeneity, identifying 8 subsets (Supplemental Figure 1C). Neu-C1, characterized by high expression of *S100a9* and *Nfkb1*, and Neu-C2, enriched for *Tnf* and *Il23a*, were predominantly enriched in the WT mice, whereas Neu-C4, marked by elevated *Apoe* and *Il10*, was more abundant in the *Card9*^–/–^ mice. (Supplemental Figure 1, D–F). Subsequent comparative analyses further revealed that multiple proinflammatory cytokines and chemokines, including *Il1b, Tnf, Ccl5, Cxcl9,* and *Cxcl10*, were downregulated in the *Card9*^–/–^ group (Supplemental Figure 1G). Gene ontology (GO) enrichment analysis consistently indicated that key pathways associated with cytokine production, ROS response, and other immune-related processes were suppressed in *Card9*^–/–^ neutrophils (Supplemental Figure 1H).

Collectively, these findings are consistent with those of previous studies, supporting the notion that CARD9 is essential for maintaining neutrophil function in antifungal immunity. Future studies may help to elucidate its detailed regulatory mechanisms.

### Exhaustion-like Th1 cells are more pronounced in Card9^–/–^ mice and correlate with antiinflammatory macrophages.

**Figure 3:**
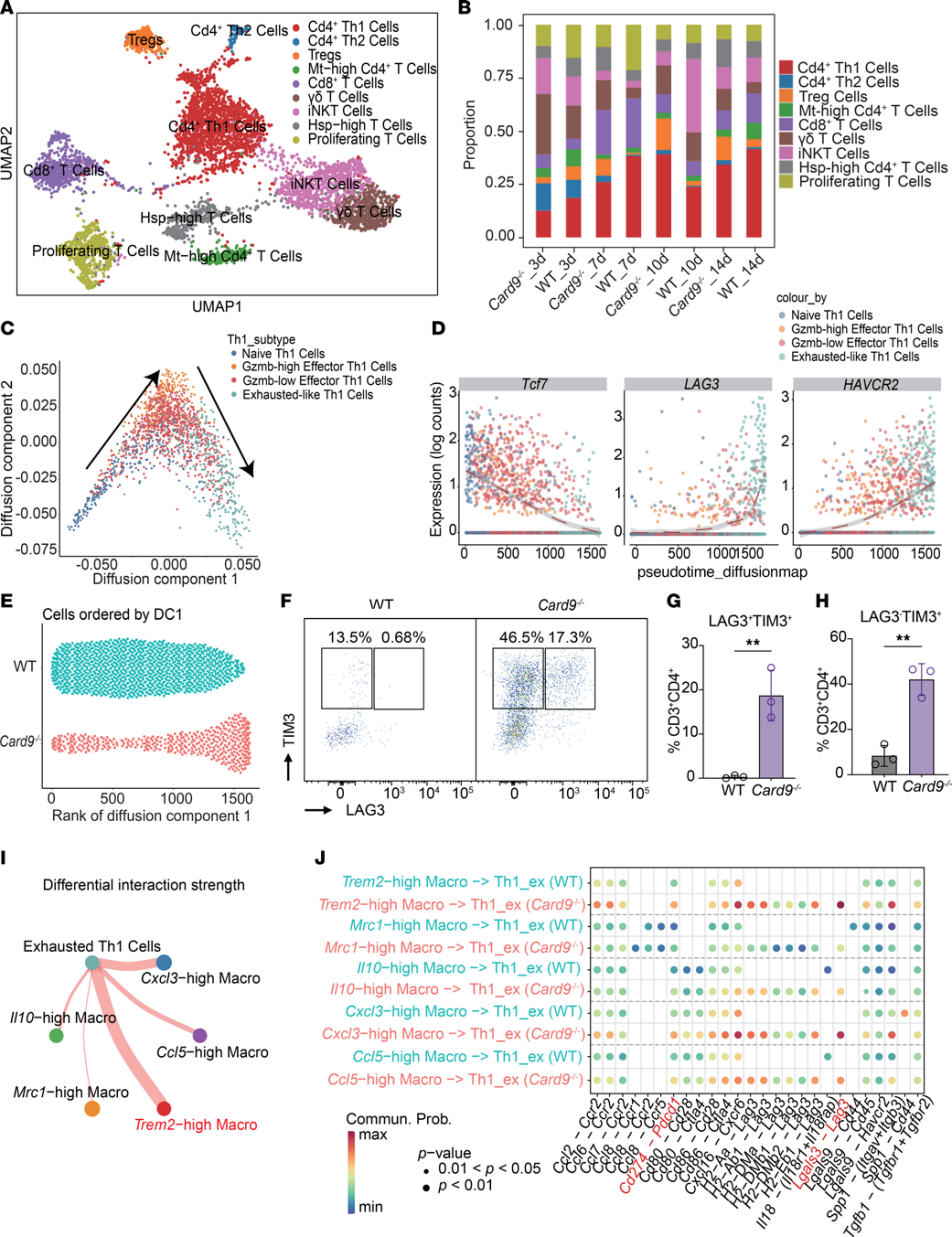
Increased abundance of exhaustion-like Th1 cells in *Card9*^–/–^ mice. (**A**) UMAP of 9 T cell subsets. Each dot represents a single cell, colored according to the specific cell type. (**B**) The stacked bar chart shows the percentage distribution of 9 T cell subsets across all samples. (**C**) The diffusion map illustrates the developmental trajectory of Th1 cells, with the direction of development indicated by arrows. This process was implemented by using the R package destiny (https://github.com/theislab/destiny). (**D**) Scatter plots show the expression of naive and exhaustion markers throughout the pseudotime of the Th1 cell development process. The dashed line represented the fitted trend of changes. Each point represented a Th1 cell, with its color corresponding to that in **C**. (**E**) Scatter plot shows the distribution of Th1 cells in the WT group and the *Card9*^–/–^ group. Each point represents a Th1 cell, with its *x*-axis corresponding to the ordinal value of diffusion component 1, arranged from smallest to largest in Supplemental Figure 3C. (**F**–**H**) Representative flow cytometry plots for TIM3 and LAG3 staining and frequency of LAG3^+^TIM3^+^CD4^+^ T cell subsets (**G**) and LAG3^–^TIM3^+^CD4^+^ T cell subsets (**H**) in murine footpad lesion at day 10 after infection. One data point denotes a result from 1 mouse. (**I**) The circle plot demonstrates the enhanced interaction strength between the major macrophage subpopulations and exhaustion-like Th1 cells in the *Card9*^–/–^ group. (**J**) The bubble plot shows the main ligand-receptor pair between the major macrophage subpopulations and exhaustion-like Th1 cells in WT and *Card9*^–/–^ group. Commun. Prob., communication probability. Data are shown as the mean ± SD. **P < 0.01 and ***P < 0.001, by 2-tailed Student’s *t* test (**G** and **H**), and pairwise Wilcoxon rank sum test (**J**).

CARD9 has been shown to regulate the activation of innate immune cells and the production of cytokines, thereby shaping adaptive T cell responses (7). Due to the altered macrophage phenotypes and reduction in T cells observed in *Card9*^–/–^ mice infected with *P*. *verrucosa*, we conducted a subpopulation analysis of T cells (Figure 3A). We found *Card9*^–/–^ mice had a higher frequency of Tregs and Th2 cells but a lower proportion of Th1 cells than did WT mice (Figure 3B and Supplemental Figure 2A).

To gain further insight into the functional status of the predominant T cell subsets, we performed a pseudotime trajectory analysis of Th1 cells (Figure 3C). The results revealed a progressive shift from a naive state through an effector phenotype and, ultimately, to an exhausted state during infection (Figure 3, C and D). Notably, *Card9*^–/–^ mice had a greater accumulation of exhaustion-like Th1 cells within the lesions on days 10 and 14 after infection (Figure 3E and Supplemental Figure 2, B and C). Flow cytometry analysis confirmed that CD4^+^ T cells in *Card9*^–/–^ murine lesions exhibited markedly higher expression of immune checkpoints than those in WT control mice on day 10 after infection (Figure 3, F–H), whereas no differences were observed prior to infection (Supplemental Figure 2D). Furthermore, mIHC revealed a greater degree of colocalization between these immune checkpoints and the CD4^+^ T cell population in lesions of *Card9*^–/–^ mice on day 10 after infection (Supplemental Figure 2, E and F).

Collectively, these results suggest CARD9 plays a critical role in regulating T cell recruitment and function during dematiaceous fungal infections. Specifically, CARD9 deficiency promotes the accumulation of immunosuppressive Treg cells and exhaustion-like Th1 cells.

Given that CARD9 is predominantly expressed in myeloid rather than lymphoid cells, the observed alteration in T cells is likely to be a secondary effect of altered myeloid cell function. The interactome analysis of primary immune cell populations revealed that macrophages exhibited the most obvious interactions with T cells (Supplemental Figure 2G). Previous studies have shown that certain macrophage subsets can promote T cell exhaustion (40, 41). We further examined the interactions between major macrophage subpopulations and exhaustion-like Th1 cells; the results indicated that TREM2^hi^ macrophages displayed higher interactions with exhaustion-like Th1 cells (Figure 3I). Additionally, we analyzed the ligand-receptor interactions between macrophage subpopulations and exhausted Th1 cells. Macrophages from the *Card9*^–/–^ group demonstrated stronger interactions with exhausted Th1 cells compared with those from the WT group. In particular, ligand-receptor pairs such as *Tgfb1*-(*Tgfbr1+Tgfbr2*), *Lgals3-Lag3*, and *Cd274-Pdcd1* exhibited a marked increase in signaling strength, suggesting these enhanced interactions may contribute to the promotion of Th1 cell exhaustion in the Card9-deficient condition (Figure 3J). These findings further support the notion that CARD9 deficiency promotes the accumulation of antiinflammatory macrophages, which may contribute to the impaired T cell responses observed in lesions of *Card9*^–/–^ mice.

### TREM2^hi^ macrophages in lesions of patients with CARD9 deficiency who have dematiaceous fungal infection.

**Figure 4:**
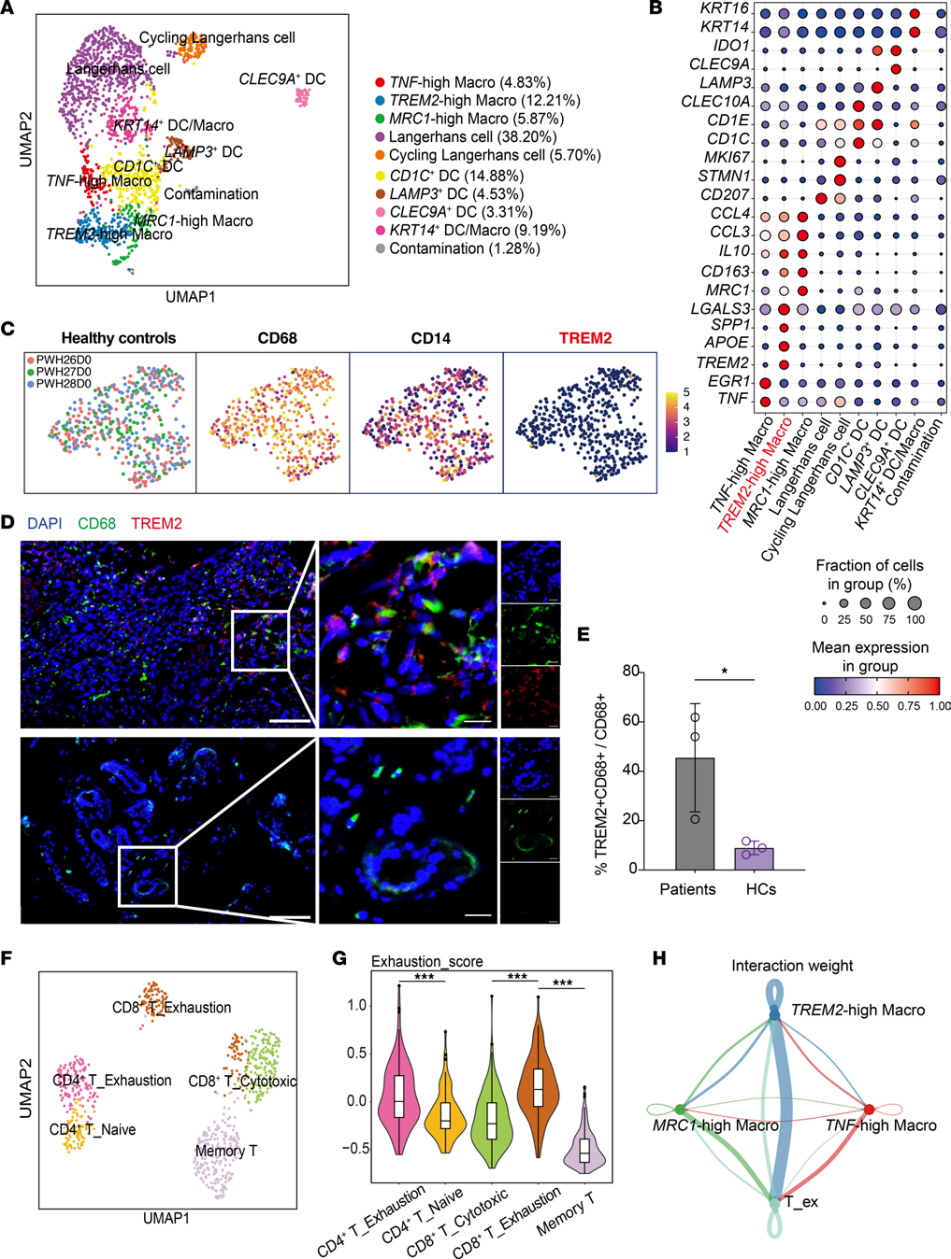
Antiinflammatory TREM2^hi^ macrophages in lesions from a patient with CARD9 deficiency and phaeohyphomycosis. (**A**) UMAP of macrophages and dendritic cells in the samples from the patient with CARD9 deficiency. Each point represents an individual cell, with the proportion of each subset within the total cell population annotated in the graph. (**B**) The bubble chart shows the mean relative expression of signature genes across the macrophage and dendritic cell subsets. (**C**) A UMAP plot shows the expression of *TREM2* in macrophages within skin tissues obtained from healthy individuals (*n* = 3). (**D** and **E**) Representative staining of TREM2^+^ macrophages (TREM2 and CD68) in lesions of patients with CARD9 deficiency and control participants. Scale bars: 100 μm (left) and 20 μm (right). The bar plots show the quantification results (**E**). One data point represents the statistical result of 1 sample (*n* = 3). HC, healthy control. (**F**) A UMAP projection displays the cellular distribution of the CD4^+^ T cell subset, CD8^+^T_*GZMK* subset, CD8^+^T_*HAVCR2* subset, and memory T cell subset as depicted in Supplemental Figure 2A; these were categorized into naive, cytotoxic, and exhaustion states based on the expression of marker genes. (**G**) Violin plots show the exhaustion score (defined by 5 genes: *LAG3*, *TIGIT*, *PDCD1*, *CTLA4*, and *HAVCR2*) of 5 T cell subsets. Box plots overlaid on the violins depict the interquartile range and median score for each subset. (**H**) The bubble chart shows the interactions between the major macrophage subsets and exhaustion-like Th1 cells in the patient with CARD9 deficiency. Data are shown as the mean ± SD. *P < 0.05 and ***P < 0.001, by 2-tailed Student’s *t* test (**E**), or by 1-way ANOVA with Tukey’s test (**G**).

Building on the murine infection model findings, we validated our observations in a patient with CARD9 deficiency who had a subcutaneous dematiaceous fungal infection. Three distinct macrophage subpopulations were identified (Figure 4A). The most abundant subset was TREM2^hi^ macrophages, whose transcriptional profile closely resembled those in murine lesions (Figure 4, A and B). In contrast, analysis of skin tissues from 3 healthy control study participants revealed minimal TREM2 expression in macrophages (Figure 4C). Further, mIHC analysis confirmed the high TREM2 expression and its colocalization with the macrophage marker CD68 in skin lesions from 3 patients with CARD9 deficiency who had dematiaceous fungal infections, compared with lesions from 3 healthy control participants (Figure 4, D and E).

T cell analysis showed Tregs were the largest CD4^+^ T cell subset (Supplemental Figure 3, A and B), with a notable proportion of the remaining CD4^+^ and CD8^+^ T cells exhibiting signs of exhaustion, characterized by a high immune checkpoint expression and elevated exhaustion scores (Figure 4, F and G). Using mIHC, we further confirmed that the immune checkpoints TIM3 and LAG3 were notably colocalized with CD4 in the skin lesions of 3 patients with CARD9 deficiency with subcutaneous dematiaceous fungal infection, in comparison with those of 3 healthy control participants (Supplemental Figure 3C). Moreover, we analyzed the receptor-ligand interactions between macrophage subsets and exhaustion-like CD4^+^ T cells. This analysis corroborated our findings in the mouse model, demonstrating that the TREM2^hi^ macrophage subset had the most prominent interactions with exhaustion-like CD4^+^ cells (Figure 4H). In conclusion, these findings strongly validate our findings in a mouse infection model, indicating that CARD9 deficiency leads to the differentiation of antiinflammatory macrophages, which may contribute to impaired T cell responses in human skin lesions of dematiaceous fungal infection.

### CARD9 deficiency induces high TREM2 expression in macrophages and impairs antifungal infection.

**Figure 5:**
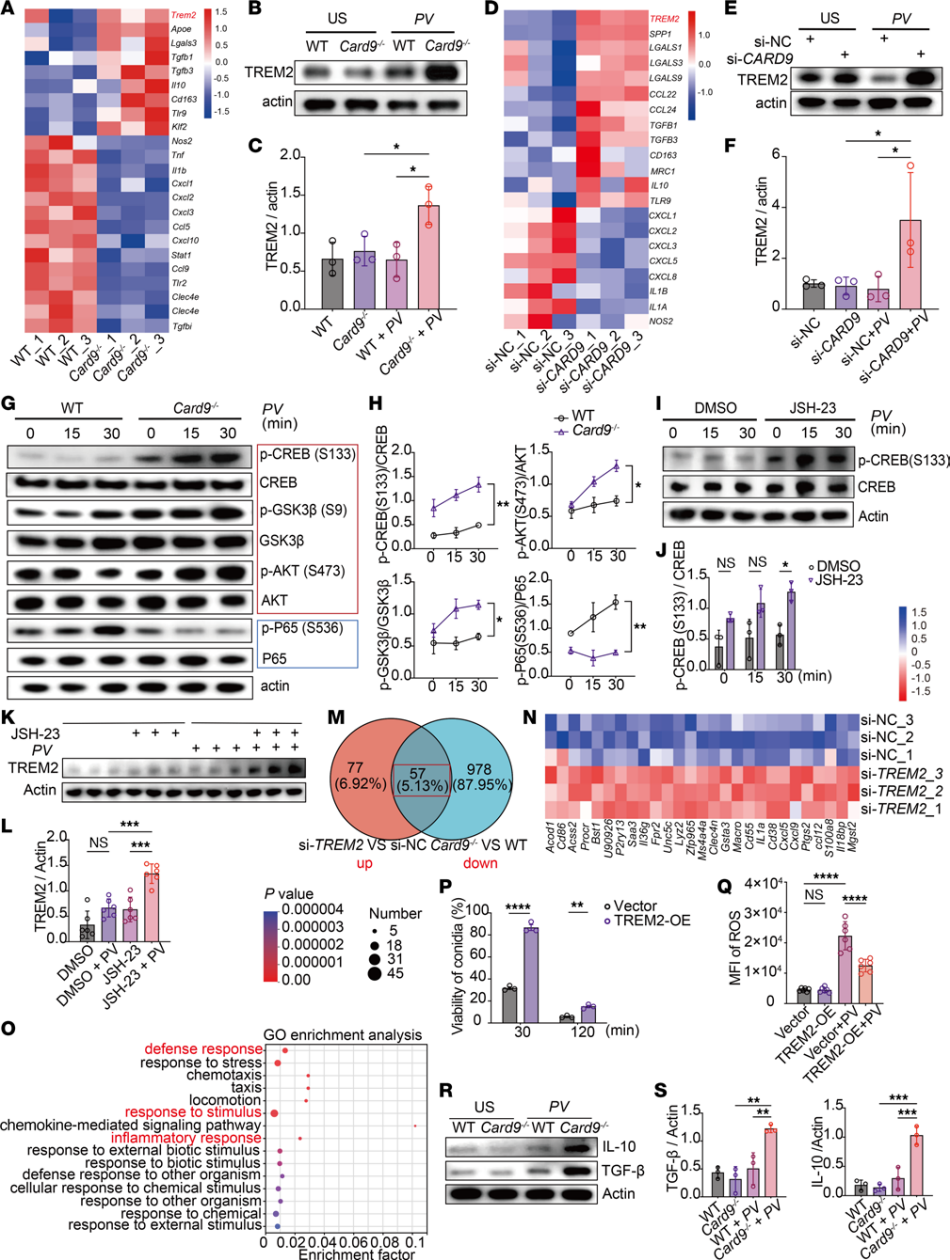
CARD9 deficiency induces higher TREM2 expression in macrophages and impairs antifungal infection. (**A**–**F**) Heatmap of selected gene expression from RNA-seq and Western blot with densitometric analysis of TREM2 in BMDMs (**A**–**C**) and THP-1 cells (**D**–**F**) stimulated with *P*. *verrucosa* for 24 hours (*n* = 3). (**G** and **H**) Western blot (**G**) and densitometric (**H**) analyses of phosphorylated and total P65, AKT, GSK3β, and CREB in BMDMs stimulated with *P*. *verrucosa*. (**I**–**L**) Western blot and densitometric analyses of phosphorylated CREB (**I** and **J**) and TREM2 (**K** and **L**) in WT BMDMs with or without JSH-23 pretreatment, after *P*. *verrucosa* stimulation. (**M**–**O**) BMDMs were transfected with a small interfering negative control (si-NC) or si-TREM2 and stimulated with *P*. *verrucosa* for 24 hours. Venn diagram (**M**) and heatmap (**N**) show the overlap between genes downregulated in *Card9*^–/–^ si-NC versus WT si-NC and those upregulated in *Card9*^–/–^ si-TREM2 versus *Card9*^–/–^ si-NC. The bubble plot shows GO enrichment of the overlap genes in *Card9*^–/–^ si-TREM2 BMDMs (**O**). (**P** and **Q**) Killing efficacy analysis (**P**) and ROS production of TREM2-overexressing RAW 264.7 cells and controls with *P*. *verrucosa* stimulation for 60 minutes (**Q**). (**R** and **S**) Western blot (**R**) and densitometric (**S**) analysis of IL-10 and TGF-β in BMDMs stimulated with *P*. *verrucosa* for 72 hours. In **A**, **D**, and **M**, columns represent replicates from independent culture wells (*n* = 3). In **C**, **F**, **H**, **J**, **L**, and **S**, each point represents an independent replicate. Data are shown as mean ± SD. *P < 0.05, **P < 0.01, ***P < 0.001, and ****P < 0.0001, by 1-way ANOVA with Tukey’s multiple-comparison test (**C**, **F**, **L**, **Q**, and **S**), 2-way ANOVA (**H**), and multiple unpaired *t* tests with Holm-Šídák correction (**J** and **P**). All stimulations used heat-killed *P*. *verrucosa* at MOI 10. OE, overexpressing; PV, *Phialophora verrucosa*; US, unstimulated.

To further determine the mechanisms underlying the accumulation of TREM2^hi^ macrophages in CARD9-deficient skin lesions, we conducted RNA-seq to analyze the transcriptional profile of bone marrow–derived macrophages (BMDMs) derived from WT and *Card9*^–/–^ mice stimulated with heat-killed *P*. *verrucosa* for 24 hours. Several genes exhibited differential expression patterns between WT and *Card9*^–/–^ BMDMs (Figure 5A). The upregulated genes in *Card9*^–/–^ BMDMs included *Trem2*, *Lgals3*, and *Apoe*, which are characteristics of the TREM2^hi^ macrophage subset identified in the in vivo infection model (Figure 5A and Figure 2D). Additionally, the expression of antiinflammatory cytokine, including *Tgfb1*, *Tgfb3*, and *Il10,* was also higher in *Card9*^–/–^ BMDMs (Figure 5A). In contrast, the upregulated genes in WT BMDMs included *Ccl5* and *Cxcl3*, which were specifically expressed in *Cxcl3*^hi^ macrophage and *Ccl5*^hi^ macrophage subsets observed in vivo (Figure 5A and Figure 2D). Furthermore, the expression of proinflammatory cytokines and chemokines, such as *Il1b, Tnf, Cxcl1*, and *Cxcl2*, was higher in WT BMDMs (Figure 5A).

Western blotting was performed to validate the differential expression of TREM2 in BMDMs. *Card9*^–/–^ BMDMs had substantially higher TREM2 levels than WT BMDMs after heat-killed *P*. *verrucosa* simulation (Figure 5, B and C). Additionally, ELISA analysis of culture supernatants revealed no difference in the levels of soluble TREM2, suggesting the increased expression observed by Western blot primarily reflected the membrane-bound form, rather than enhanced secretion (Supplemental Figure 4A). Similarly, the knockdown of endogenous CARD9 in THP-1 cells resulted in consistent differential expression patterns. RNA-seq and immunoblotting confirmed *P*. *verrucosa–*induced higher expression of TREM2 in CARD9-knockdown THP-1 cells (Figure 5, D–F).

Subsequently, we conducted a Kyoto Encyclopedia of Genes and Genomes (KEGG) enrichment analysis to compare the activation of *P*. *verrucosa*–induced signaling molecules in WT and *Card9*^–/–^ BMDMs. These results demonstrated markedly diminished activation of NF-κB signaling in *Card9*^–/–^ BMDMs (Supplemental Figure 4B). By contrast, activation of the PI3K/AKT signaling pathway was greater in *Card9*^–/–^ BMDMs than in WT BMDMs (Supplemental Figure 4B), suggesting CARD9 played a pivotal role in regulating the equilibrium between NF-κB and PI3K/AKT signaling pathways.

Previous studies revealed that TLRs trigger antiinflammatory signaling via the PI3K/AKT/GSK3β pathways in macrophages, which converge to activate CREB (42, 43). The relative amounts of active nuclear CREB and NF-κB p65 determine subsequent association with the nuclear coactivator CBP/p300, thereby regulating the proinflammatory and antiinflammatory responses in macrophages (43, 44). To elucidate whether the antiinflammatory phenotype of *Card9*^–/–^ BMDMs is associated with alterations in NF-κB and PI3K/AKT signaling, we conducted immunoblotting assays to measure the activation of key molecules in these pathways. Upon *P*. *verrucosa* stimulation, *Card9*^–/–^ BMDMs had lower phosphorylation of the NF-κB p65 subunit and higher phosphorylation of Akt, GSK3β, and CREB in comparison with WT BMDMs (Figure 5, G and H). Furthermore, pretreatment of WT BMDMs with NF-κB inhibitor followed by *P*. *verrucosa* stimulation resulted in a notable increase in the phosphorylation of CREB (Figure 5, I and J, and Supplemental Figure 4C), as well as the expression of TREM2 (Figure 5, K and L). These findings provide further evidence that, in conditions of impaired NF-κB signaling, CREB is activated and contributes to the transcriptional upregulation of TREM2.

To further elucidate the role of TREM2 upregulation in CARD9-deficient macrophage, we performed transcriptome sequencing of WT and *Card9*^–/–^ BMDMs following TREM2 knockdown and 24-hour *P*. *verrucosa* stimulation. Notably, a subset of genes downregulated in *Card9*^–/–^ BMDMs exhibited increased expression upon TREM2 knockdown compared with WT BMDMs (Figure 5M). These included proinflammatory factors, such as *Il1α, Cxcl5,* and *Cxcl9,* as well as the antimicrobial peptide *S100A8* (Figure 5N). GO functional enrichment analysis revealed that the upregulated genes in the small interfering TREM2 (si-TREM2) group were enriched in biological processes, including defense response, response to stimulus, and inflammatory response (Figure 5O). Furthermore, KEGG pathway enrichment analysis indicated marked enrichment of upregulated genes in cytokine-cytokine receptor interactions and signaling pathways related to IL-17, TNF, and chemokines (Supplemental Figure 4D). To more directly assess the functional role of TREM2^hi^ macrophages in antifungal immunity, we overexpressed TREM2 in RAW264.7 cells (Supplemental Figure 4, E and F). In comparison with control cells, TREM2-overexpressing macrophages demonstrated impaired fungicidal activity (Figure 5P) and decreased production of total ROS (Figure 5Q).

Single-cell interaction analysis suggested TREM2^hi^ macrophages may contribute to T cell exhaustion under Card9-deficient conditions (Figure 3, I and J). Notably, *Card9*^–/–^ BMDMs exhibited elevated expression of *Il10* and *Tgfb1*, 2 immunoregulatory cytokines implicated in driving T cell exhaustion (41, 45, 46). Consistent with the transcriptional data, Western blot analysis confirmed increased protein levels of IL-10 and TGF-β in *Card9*^–/–^ BMDMs under *P*. *verrucosa* stimulation (Figure 5, R and S), providing further support for a *Card9*^–/–^ macrophage–mediated mechanism promoting T cell exhaustion.

Collectively, these data indicate that in CARD9-deficient macrophages, *P*. *verrucosa*–induced NF-κB signaling activation is constrained, leading to enhanced activation of CREB and the predominance of Trem2^hi^ macrophages. Moreover, upregulation of TREM2 expression in CARD9-deficient hosts is associated with impaired innate and adaptive antifungal function.

### CARD9 negatively regulates Trem2 expression by activating C/EBPβ.

**Figure 6:**
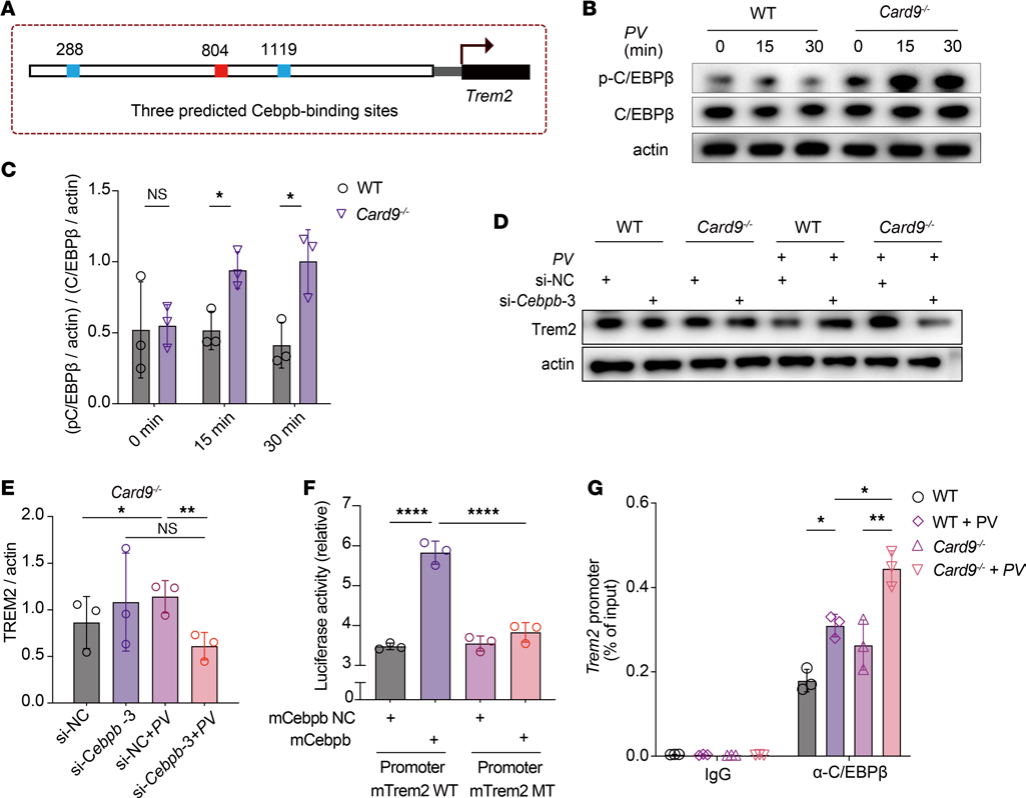
CARD9 negatively regulates *Trem2* expression by activating C/EBPβ. (**A**) Promoter region of WT *Trem2*, showing 3 predicted C/EBPβ-binding sites at positions. (**B** and **C**) Western blot (**B**) and densitometric (**C**) analysis of phosphorylated (p-) and total C/EBPβ (left margin) in BMDMs isolated from WT and *Card9*^–/–^ mice and stimulated for 0–30 minutes (above lanes) with heat-killed *P*. *verrucosa* conidia (MOI 10). Each data point represents an independent experimental replicate (*n* = 3). (**D** and **E**) Knockdown of endogenous C/EBPβ by RNA interference in BMDMs, which were transfected with siRNA against murine C/EBPβ and nontargeting control siRNA using Lipofectamine 3000 transfection reagent (Thermo Fisher). Cells were cultured for 48 hours after transfection and then stimulated with heat-killed *P*. *verrucosa* conidia (MOI 10) for 24 hours. Cell lysates were subjected to Western blot analysis using indicated antibodies (**D**) and then quantified using densitometric analysis in *Card9*^–/–^ group (**E**). Each data point represents an independent experimental replicate (*n* = 3). (**F**) Firefly luciferase activity in human embryonic kidney 293T cells cotransfected with constructs for the overexpression of Cebpb and a construct containing various *Trem2* promoter–driven firefly luciferase constructs together with an EF1α promoter–driven *Renilla* luciferase reporter; results were normalized to those of *Renilla* luciferase. Ctr, control construct lacking Cebpb cotransfected with a construct containing the *Trem2* promoter. (**G**) Chromatin immunoprecipitation (with control IgG or anti-Cebpb) and PCR analysis of the binding of Cebpb to the *Trem2* promoter in BMDMs obtained from WT mice and *Card9*^–/–^ mice and left unstimulated or challenged for 4 hours in vitro with heat-killed *P*. *verrucosa* spores (MOI 10). Data are shown as the mean ± SD. *P < 0.05 and **P < 0.01, and ****P < 0.0001, by multiple unpaired *t* tests with Holm-Šídák correction (**C**), or 1-way ANOVA with Tukey’s multiple-comparison test (**E**–**G**). PV, *Phialophora verrucosa*; si-NC, small interfering negative control; mTrem2, mouse Trem2; MT, mutant type.

To further elucidate the mechanism by which the CARD9-related pathway regulates the expression of TREM2, we screened, using bioinformatics analysis, transcription factors that can directly bind to the *Trem2* promoter in both mouse and human cells; this ultimately led to the identification of C/EBPβ as the most promising candidate (Figure 6A). Activated CREB promotes the expression and activation of C/EBPβ. Western blot analysis was performed to confirm the increased activation of C/EBPβ in *Card9*^–/–^ BMDMs upon *P*. *verrucosa* stimulation in comparison with WT BMDMs (Figure 6, B and C). Previous studies also demonstrated that the activation of C/EBPβ can promote the antiinflammatory polarization of macrophages (44, 47).

To ascertain whether C/EBPβ can regulate *P*. *verrucosa*–induced expression of TREM2 in macrophages, siRNA was used to knockdown C/EBPβ in BMDMs, which were then stimulated with *P*. *verrucosa*. Knockdown of C/EBPβ notably suppressed *P*. *verrucosa*–induced expression of TREM2 in *Card9*^–/–^ BMDMs (Figure 6, D and E). To determine whether C/EBPβ directly dictates the *Trem2* transcription, we also used a dual-luciferase reporter assay, which revealed that C/EBPβ overexpression in 293T human embryonic cells resulted in *Trem2* promoter activation (Figure 6F). Similarly, we conducted chromatin immunoprecipitation assays and observed C/EBPβ binding to the promoter region of *Trem2* in CARD9-deficient macrophages (Figure 6G). These results collectively indicate that augmented C/EBPβ signaling in CARD9-deficient macrophages can directly bind to the promoter region of *Trem2*, thereby exerting a positive regulatory effect on its expression.

### Anti-TREM2 antibody improves the antifungal immune response in vivo and in vitro.

**Figure 7:**
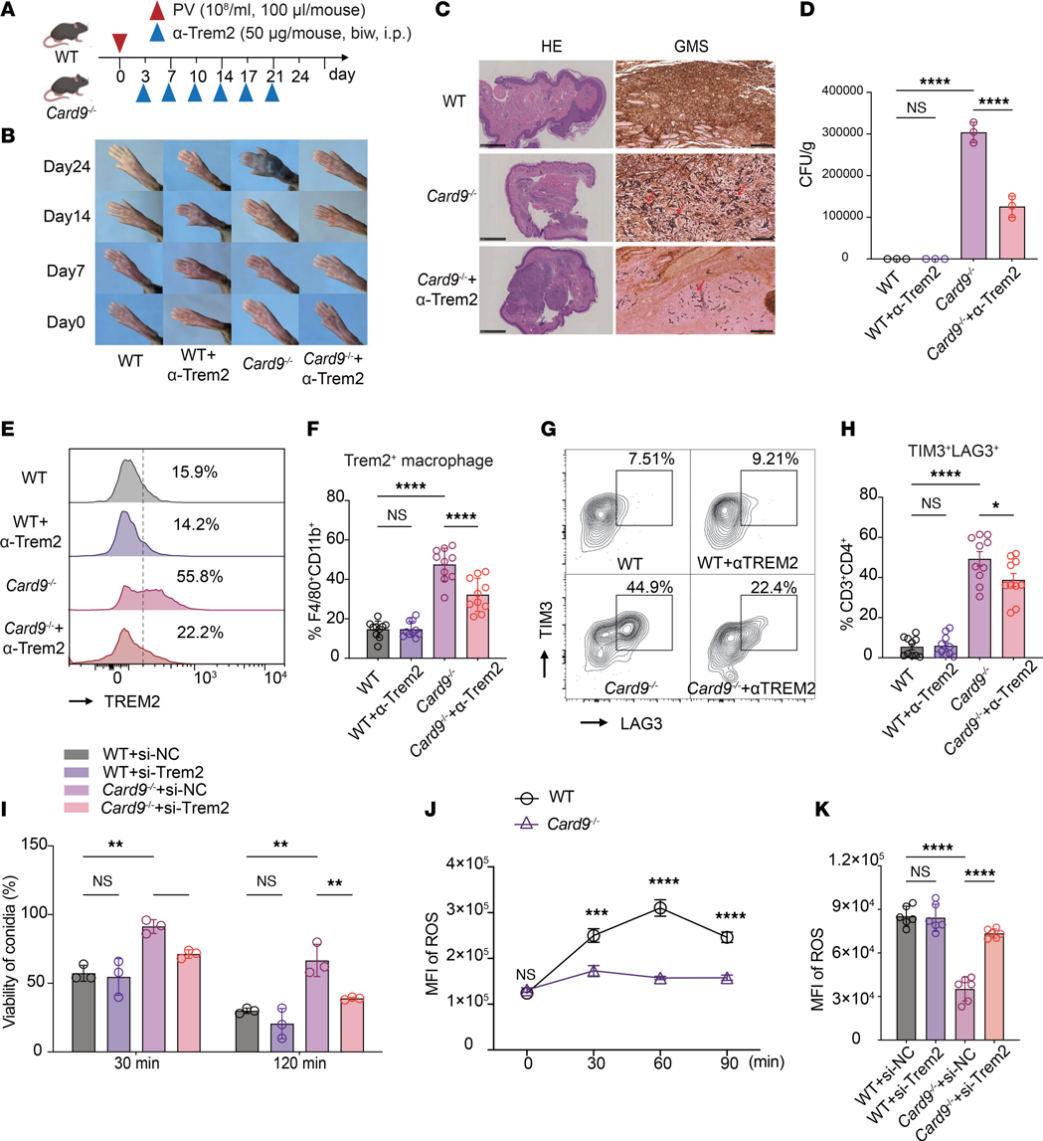
Anti-TREM2 antibody improves the antifungal immune response in vivo and in vitro. (**A** and **B**) WT and *Card9*^–/–^ mice were subcutaneously injected with *P*. *verrucosa* and treated intraperitoneally with PBS or anti-TREM2 antibody. Experimental scheme (**A**) and natural course (**B**) of infection. (**C**) Histopathology of H&E and Grocott’s methenamine silver (GMS) staining of footpads from infected mice at day 14 after infection. Scale bars: 1 mm (H&E stain) and 100 μm (GMS stain). Arrows indicate fungal yeast and hyphae. (**D**) Fungal burden of footpad from WT and *Card9*^–/–^ mice on day 14 after infection. (**E** and **F**) Representative flow cytometry histogram plots for TREM2 staining (**E**) and frequency of TREM2^+^ macrophage subsets (**F**) in murine lesions. One data point denotes a result from 1 mouse (*n* = 10, from 3 independent experiments). (**G** and **H**) Representative flow cytometry contour plots for TIM3 and LAG3 staining (**G**) and frequency of TIM3^+^ LAG3^+^CD4^+^ T cell subsets (**H**) in murine lesions. One data point denotes a result from 1 mouse (*n* = 10, from 3 independent experiments). (**I**–**K**) Knockdown of endogenous TREM2 by RNA interference in BMDMs. Cells were cultured for 48 hours after transfection and then stimulated with heat-killed *P*. *verrucosa* spores (MOI 10) for the indicated times. (**I**) Killing efficacy analysis of BMDMs of *Card9*^–/–^ mice. (**J** and **K**) Total ROS production of WT and *Card9*^–/–^ BMDMs at the indicated time point was measured by the Reactive Oxygen Assay kit (Beyotime). Data in **B**, **C**, and **I**–**K** are representative of 3 independent experiments. Data are shown as the mean ± SD. *P < 0.05, **P < 0.01, ***P < 0.001, and ***P < 0.001, by 1-way ANOVA with Tukey’s multiple-comparison test (**D**, **F**, **H**, **I**, and **K**), or 2-way ANOVA with Šídák’s multiple-comparison test (**J**). PV, *Phialophora verrucosa*; NC, negative control.

To investigate the potential therapeutic benefits of targeting TREM2 in the treatment of phaeohyphomycosis, WT and *Card9*^–/–^ mice were infected with *P*. *verrucosa* and treated with a blocking antibody of TREM2 (Figure 7A). The administration of anti-TREM2 antibody delayed disease progression and reduced the footpad swelling rate in *Card9*^–/–^ mice compared with that in the control group (Figure 7B). However, no differences in footpad lesion phenotypes were observed between the antibody treatment and control groups in WT mice (Figure 7B). Histological examination of the footpad on day 21 after infection showed a marked reduction in inflammatory cell infiltration, smaller infectious granulomas, and a lower fungal burden (i.e., spores and hyphae) in the lesions of *Card9*^–/–^ mice treated with anti-TREM2 antibody compared with that in nontreated *Card9*^–/–^ mice (Figure 7C). Furthermore, treatment with an anti-TREM2 antibody resulted in a notable reduction in local fungal loads in *Card9*^–/–^ mice on day 21 after infection (Figure 7D).

Flow cytometry was performed to elucidate the cellular mechanisms underlying anti-TREM2 antibody treatment. In WT mice, no differences were observed in the proportions of different macrophage subsets between the antibody-treated and control groups. However, in *Card9*^–/–^ mice, the anti-TREM2 antibody treatment group had a substantially lower proportion of TREM2^+^ antiinflammatory macrophages than the control group (Figure 7, E and F). Pretreatment of TREM2-overexpressing RAW 264.7 cells with the blocking antibody did not affect subsequent detection by flow cytometry, confirming no epitope interference (Supplemental Figure 4E). Furthermore, the anti-TREM2 antibody–treated group displayed reduced expression of immune checkpoints (TIM3 and LAG3) on CD4^+^ T cells compared with the nontreated group. In contrast, both treatment and control groups of WT mice exhibited minimal expression of immune checkpoints on CD4^+^ T cells within the lesions (Figure 7, G and H). These results indicate anti-TREM2 antibody may serve as a potential therapeutic strategy for enhancing host innate and adaptive immunity against fungal infections in *Card9*^–/–^ mice.

Previous studies have demonstrated that CARD9-deficient macrophages exhibit defects in killing *P*. *verrucosa* (48). To ascertain whether targeting TREM2 affects the fungal-killing ability of macrophages in vitro, siRNA was used to knock down the expression of TREM2. The knockdown of endogenous TREM2 restored the spore-killing ability of *Card9*^–/–^ BMDMs, whereas it had no effect on WT BMDMs (Figure 7I). Moreover, the generation of ROS is a pivotal effector mechanism of macrophages in antifungal immunity. HALLMARK gene set scoring among the major macrophage subsets indicated the TREM2^hi^ macrophage subsets had a reduction in ROS pathway function (Figure 2E), and overexpression of TREM2 in RAW 264.7 cells also showed impaired ROS production (Figure 5Q). Consequently, we investigated the impact of targeting TREM2 on total ROS production in macrophages. *Card9*^–/–^ BMDMs exhibited a markedly diminished capacity to generate ROS upon stimulation with *P*. *verrucosa* compared with WT BMDMs (Figure 7J). Importantly, the knockdown of TREM2 considerably enhanced the ROS generation by *Card9*^–/–^ BMDMs (Figure 7K).

These results provide further evidence for the crucial role of the TREM2-mediated antiinflammatory pathway in compromising antifungal immunity in the context of CARD9 deficiency. Modulation of TREM2 signaling represents a promising strategy to enhance the fungicidal ROS response and fungal killing by macrophages and improve host defense against dematiaceous fungal infections in CARD9-deficient settings.

## Discussion

Fungal infections have been increasing globally, presenting a considerable disease burden and endangering public health. Over the past decades, our understanding of the mechanisms underlying the host antifungal immune response deepened but remains inadequately delineated (1). To elucidate how the immune system responds to fungal skin infections, we established a subcutaneous *P*. *verrucosa* infection model. Using high-throughput scRNA-seq, we generated a detailed global portrait of the local immune cell populations in infectious skin lesions and conducted preliminary validation using lesions obtained from patients. This single-cell atlas of the antifungal immune response establishes a crucial foundation for future investigations into the immunological mechanisms underlying fungal diseases, advancing both our fundamental understanding and the potential for targeted immunotherapeutic strategies.

As a key adaptor protein downstream of fungal pattern recognition receptors, CARD9 efficiently integrates recognition signals from multiple receptors and regulates host antifungal immunity (7). CARD9 deficiency notably increases susceptibility to various fungal infections (49–52). To further evaluate the role of CARD9 in host antifungal immunity, we conducted a comparative analysis of immune cells in skin lesions of WT and *Card9*^–/–^ mice. The overall number of local macrophages were not markedly different between the groups, suggesting CARD9 does not affect macrophage recruitment in *P*. *verrucosa* subcutaneous infection. However, we observed a notable alteration in macrophage phenotype. In both mice and patients with CARD9 deficiency, skin lesions exhibited a pronounced increase in the antiinflammatory TREM2^hi^ macrophage subset, whereas proinflammatory macrophages were markedly diminished. TREM2 is a crucial receptor expressed on the surfaces of macrophages and other myeloid cells (19). It mediates diverse downstream signaling pathways upon binding to various ligands, including lipids, β-amyloid peptides, TDP-43, APOE, and galectin-3, among others. Previous studies have demonstrated that TREM2 signaling promotes an antiinflammatory, tissue-repairing phenotype in macrophages, which can be detrimental to antimicrobial immunity (21, 22, 24, 25). TREM2 also suppresses the release of inflammatory mediators by negatively regulating TLR signaling during bacterial infections (52). However, the role of TREM2 in fungal infections has not been well studied. Our findings emphasize the crucial role of CARD9 signaling in regulating the equilibrium between the pro- and antiinflammatory macrophage phenotypes. Previous research has also proposed that CARD9 mediates the induction of a proinflammatory M1 phenotype by β-glucan, and loss of CARD9 promotes an antiinflammatory M2 macrophage polarization, impairing antifungal functions (17, 53). A recent study demonstrated that monocytic responses act as key protective effectors in chronic central nervous system candidiasis, showing that CARD9 deficiency impairs the early upregulation of activation markers on mononuclear phagocytes (18). In this study, we demonstrated that Card9 deficiency markedly alters the phenotype and function of monocyte-derived macrophages in a subcutaneous dematiaceous fungal infection model. Collectively, these findings underscore the critical importance of CARD9 in shaping monocyte and monocyte-derived cell plasticity and function across diverse fungal infection models.

Nonetheless, the present study has limitations. Lineage-tracing and fate-mapping strategies were not used to directly determine the developmental origin of macrophage subsets; thus, definitive evidence cannot be provided that the TREM2^hi^ population is monocyte derived. However, by integrating previously reported gene signatures of skin monocyte–derived macrophages, it was found that the transcriptional profile of the TREM2^hi^ subset closely aligns with this lineage (39). Furthermore, our in vitro experiments using BMDMs, RAW264.7 cells, and THP-1–derived macrophages consistently demonstrated that CARD9 regulates TREM2 expression and functional programs in monocyte-derived macrophages.

CARD9 is a critical mediator that links innate and adaptive immunity, and CARD9 deficiency impairs T cell differentiation. In this study, we observed a notable reduction in local T cell infiltration within the lesions of *Card9*^–/–^ mice compared with those in WT control mice. Additionally, there was an increased frequency of regulatory Tregs and Th2 cells and a lower proportion of Th1 cells, indicating CARD9 plays a crucial role in mobilizing adaptive T cell responses against fungal infection. Furthermore, we revealed the development of an exhaustion-like phenotype in both CD4^+^ and CD8^+^ T cells within fungal infection lesions, characterized by elevated inhibitory receptor expression, which was more pronounced in *Card9*^–/–^ mice. T cell exhaustion is typically associated with inadequate control and progression of chronic infections (54, 55). However, its role in fungal infections remains poorly understood, with only a few studies demonstrating increased expression of immune inhibitory receptors in systemic fungal infections (56–58). This study provides the evidence of exhaustion-like changes in T cells during cutaneous dematiaceous fungal infections. Analysis of cellular interactions indicated the interplay between antiinflammatory macrophages and exhaustion-like Th1 cells may be 1 reason for the induction of exhaustion-like changes in Th1 cells. Card9-deficient macrophages expressed higher levels of immune checkpoint ligands and secreted increased amounts of IL-10 and TGF-β, suggesting a potential mechanism by which they contribute to Th1 cell dysfunction (41, 45, 46). Further investigation is required to elucidate the underlying mechanisms and the impact of exhausted T cells on antifungal immunity.

The regulatory mechanisms governing TREM2 expression remain unclear. Previous research has shown that proinflammatory stimuli, such as LPS and IFN-γ, can downregulate TREM2 expression in macrophages (21). This likely results from the activation of the NF-κB signaling pathway, which promotes miR-34a expression, binding and suppressing the transcriptional activity of TREM2 (59). This study elucidated the potential mechanism by which CARD9 regulates the expression of TREM2 in macrophages upon fungal stimulation. We provided evidence that CARD9 likely controls TREM2 expression by regulating the balance between NF-κB/P65 and the CREB-C/EBPβ signaling pathways. CARD9 deficiency appeared to induce higher activation of CREB-C/EBPβ signaling, which positively regulated the transcriptional programming that drives the antiinflammatory TREM2^hi^ macrophage phenotype. This insight into CARD9-dependent regulation of TREM2 expression in macrophages advances our understanding of how CARD9 orchestrates the local immune response against fungal infections.

The findings suggest targeting TREM2 may enhance antifungal immunity, particularly in CARD9-deficient hosts. TREM2 has been identified as an important target for treating neurodegenerative and infectious diseases and cancer immunotherapy (19). TREM2 knockdown promotes the clearance of bacterial infections and improves T cell responses in cancer immunotherapy (60, 61). In this study, we demonstrated that modulation of TREM2 partially corrected the antiinflammatory phenotype of CARD9-deficient macrophages in response to fungal stimulation, enhancing their fungicidal activity and ROS generation. Furthermore, this intervention also alleviated exhaustion-like changes observed in Th cells, delaying infection progression in *Card9*^–/–^ mice with dematiaceous fungal infection. These findings highlight the potential of exploiting the TREM2 pathway as an adjunct immunotherapeutic strategy against CARD9-related phaeohyphomycosis. Further investigations are needed to fully elucidate the underlying mechanisms and assess the feasibility of developing TREM2-targeted therapies for clinical management. Although the present findings provide valuable insights into the host response to phaeohyphomycosis, it is acknowledged that different fungal pathogens and different infection routes may trigger distinct immune responses and regulatory mechanisms. Therefore, broader implications of TREM2^hi^ macrophages in various fungal infections demand further elucidation.

In conclusion, this study unveils the mechanism by which CARD9 regulates macrophage phenotype and antifungal function by balancing the CREB-C/EBPβ/NF-κB signaling pathway. Furthermore, we demonstrated the detrimental impact of TREM2^hi^ macrophages on host antifungal innate and adaptive immunity, highlighting their potential as therapeutic targets.

## Methods

### Sex as a biological variable.

In mouse studies, mostly male mice were used, with sex and age matched across different groups. For human studies, data were collected from both men and women.

### Construction of subcutaneous dematiaceous fungal infection model.

WT and *Card9*^–/–^ mice were injected subcutaneously in both hind footpads with 100 μL of viable *P*. *verrucosa* (1 × 10^8^ particles/mL). Starting on day 3 after infection, mice received intraperitoneal injections of an anti-TREM2 antibody (50 μg per injection, twice weekly). Fungal burden in the infected footpads was determined by plating serially diluted footpad homogenates on Sabouraud’s agar (BD Biosciences).

### Anti-Trem2 antibody expression and purification.

The anti-Trem2 antibody (clone 37012) was constructed and expressed in house. For protein expression, plasmids were mixed with PEI MAX in Freestyle 293 medium at a mass ratio of 1:4. The mixture was used to transiently cotransfect human embryonic kidney 293F cells. After 6 days of transfection, the supernatant was collected and passed through a 0.22 μM filter. The protein was purified by protein A–Sepharose column according to the manufacturer’s instructions (Repligen Corp.) and analyzed by reducing and nonreducing SDS-PAGE.

### Single-cell preparation from skin tissue.

Skin biopsy specimens were disassociated using Dispase II (Sigma-Aldrich) to separate the epidermis and dermis. The minced epidermis was further digested with 0.25% trypsin-EDTA (Gibco, Thermo Fisher Scientific) for 30 minutes and filtered with a 70 μm cell strainer (Falcon). The dermis was digested with 1 mg/mL Collagenase P (MilliporeSigma) and 100 μg/mL DNase I (MilliporeSigma) for 50 minutes and filtered using a 70 μm cell strainer (Falcon). Barcode labeling of single cells and library construction were performed using a 10× chromium system (10× genomics). The constructed library was sequenced using the Illumina Novaseq 6000 system.

### Calculating cell state scores.

To elucidate the functional states and underlying biological processes of the cells within our integrated scRNA-seq dataset, we leveraged the AddModuleScore function in the Seurat package in R. AddModuleScore calculates the score for each cell based on the expression of predefined genes, effectively summarizing the activity of the pathway or module within that cell. The predefined gene sets used in this study are specifically annotated in relevant sections of this report.

### Cell-cell contact analysis.

CellChat (version 1.6.0) was used to explore communication networks among cell populations, focusing on known ligand-receptor pairs. This analysis aimed to uncover the signaling pathways that mediate interactions between cells, gaining insights into the regulatory mechanisms that underpin cellular cooperation and coordination in our system of interest (62).

### Detection of ROS production.

We measured ROS production as previously described (12). Briefly, BMDM cells were washed with PBS twice and incubated with serum-free DMEM containing 10 μM 2′,7′-dichlorodihydrofluorescein diacetate at 37°C for 30 minutes. The cells were gently washed thrice and infected with heat-killed *P*. *verrucosa* (MOI 10) at different time points. The relative amount of ROS generated was detected using a BD FACS flow cytometer, and the MFI in the FITC channel was calculated using FlowJo, version 10.4, software.

Additional details on methods can be found in the Supplemental Methods.

### Statistics.

Data were analyzed using GraphPad Prism, version 9.0, software and are presented as the mean ± SD. Comparisons between the 2 groups were performed using a 2-tailed Student’s *t* test or pairwise Wilcoxon rank-sum test. For comparisons among multiple groups, 1-way ANOVA followed by Tukey’s post hoc test was used to determine the statistical significance. Two-way ANOVA was performed to assess the differences in footpad swelling changes over time between the 2 groups. Statistical significance was determined based on *P* values; these are reported in the figure legends where applicable.

### Study approval.

All mouse experiments were conducted in accordance with the guidelines of the Institutional Ethics Committee of Peking University First Hospital. All patients provided written informed consent before participation.

### Data availability.

The raw scRNA-seq data reported in this article have been deposited in the Genome sequence Archive in National Genomics Data Center, China National Center for Bioinformation/Beijing Institution of Genomics, Chinese Academy of Sciences (accession no. CRA028974; https://ngdc.cncb.ac.cn/gsa/browse/CRA028974 and HRA012858 https://ngdc.cncb.ac.cn/gsa-human/browse/HRA012858). Values for all data points in graphs are reported in the Supporting Data Values file.

## Author contributions

XW, FB, and WW conceptualized this study. LZ, YZ, WL, HJ, KL, and YM conducted the experiments and acquired data. LZ and ZT analyzed the data and wrote the manuscript. XW, FB, and WW edited the manuscript. LY provided reagents for this study. YXF and RL provided guidance for this study. RL and XW provided funding.

## Funding support

- National Key Research and Development Program of China (grants 2022YFC2504800 and 2022YFC2504602).
- National Natural Science Foundation of China (grants 82273543, 82030095, 82241230, and 82341007).
- Beijing Nova Program (grant 20230484339).
- National Science Fund for Distinguished Young Scholars (grant T2125002).
- Beijing Natural Science Foundation (grant Z220014).
- Clinical Medicine Plus X-Young Scholars Project of Peking University (grant PKU2025PKULCXQ013).

## Supplementary Material

Supplemental data

Unedited blot and gel images

Supporting data values

## Version 1 12/02/2025

In-Press Preview

## Version 2 02/02/2026

Electronic publication
