# Supplementary material for: CARD9-dependent macrophage plasticity regulates effective fungal clearance
Source: J Clin Invest. 2025 Dec 2;136(3):e188827. doi: 10.1172/JCI188827 (PMC12867132; doi:10.1172/JCI188827)
Supplement: Supplemental data [file jci-136-188827-s296.pdf]

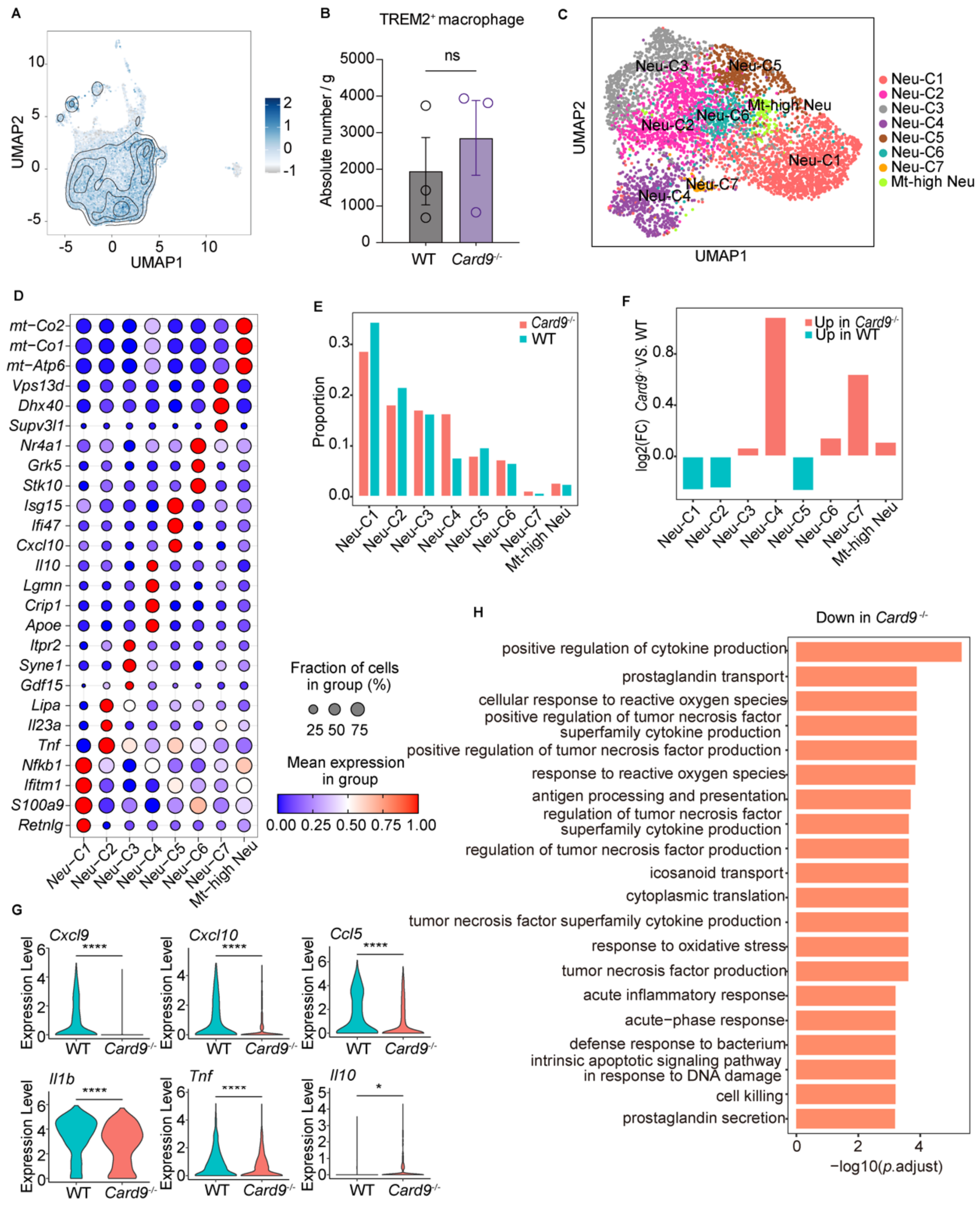

Supplementary Figure 1. CARD9-deficient neutrophils exhibit accelerated senescence and diminished proinflammatory cytokine production

(A) Overlay of the conserved classical Monocyte mean signature (1) in myeloid cells.

(B) Absolute number of TREM2^{+} macrophages in uninfected footpad tissues from WT and *Card9*$^{-/-}$ mice. (n = 3).

(C) UMAP projection of eight neutrophil subsets. Each dot represented a single cell, colored according to the specific cell type.

(D) Bubble chart shows mean relative expression of signature genes across the neutrophil subsets.

(E) The bar chart shows the proportion of eight neutrophil subsets in *Card9*$^{-/-}$ and WT groups.

(F) The bar chart displayed the log2 values of the FC (fold change) of eight neutrophil subsets proportions in the *Card9*$^{-/-}$ group relative to the WT group.

(G) Violin plots show the expression of *Cxcl9, Cxcl10, Ccl5, Il1b, Tnf*, and *Il10* in *Card9*$^{-/-}$ and WT groups.

(H) Bar plot illustrating Gene Ontology (GO) enrichment analysis of genes upregulated in neutrophils from *Card9*$^{-/-}$ group.

Data are shown as the mean ± SEM (A). ns $P > 0.05$, *$P < 0.05$, ****$P < 0.0001$, by two-tailed Student's t test (B), and pairwise Wilcoxon rank sum test (G).

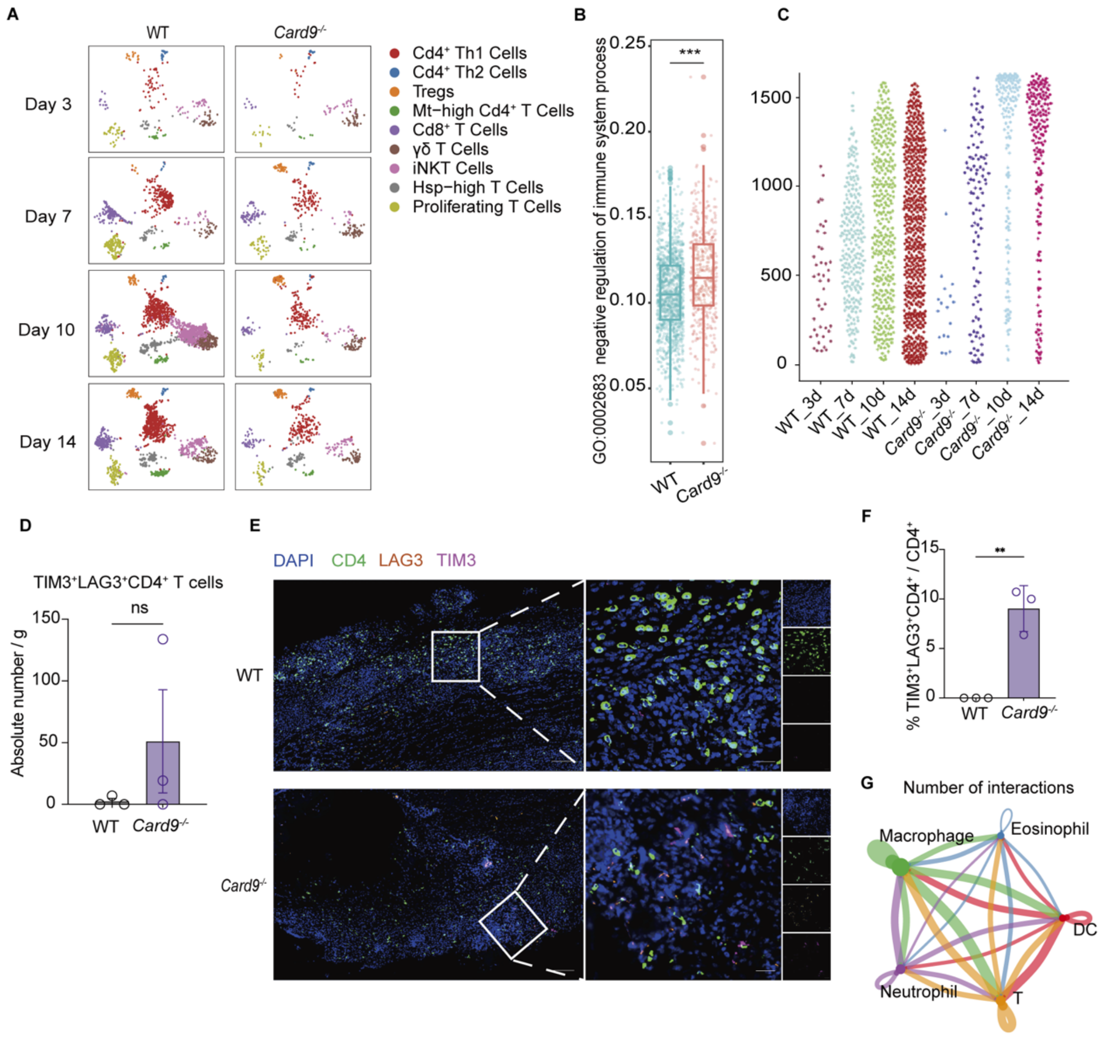

Supplementary Figure 2. Increased exhausted-like Th1 cells in *Card9*$^{-/-}$ mice

(A) The UMAP plot shows the distribution of nine T cell subsets across all samples.

(B) Box plot demonstrated the differences in the GO:0002683 pathway between the Th1 cells in two groups. Scores were calculated using the AddModuleScore function from the Seurat package. A pairwise Wilcoxon rank sum test was performed.

(C) Scatter plot shows the distribution of Th1 cells in the WT group and the *Card9*$^{-/-}$ group at all timepoints. Each point represented a Th1 cell.

(D) Absolute number of TIM3^{+} LAG3^{+} macrophages in uninfected footpad tissues from WT and *Card9*$^{-/-}$ mice. (n = 3).

(E and F) Co-localization of $CD4^+$ and immune checkpoint (TIM3 and LAG3) in murine footpad lesion at day 10 post-infection. Scale bars, 100 μm (left) and 20 μm (right). The bar plots show the quantification results (F). One data point represents the statistical result of one field of view (n = 3 fields analyzed per condition).

(G) The circle plot indicates the number of cell-cell interactions among the major cell populations, with line thickness directly proportional to the interaction frequency.

Data are shown as the mean ± SEM. $^{**}P < 0.01$, $^{***}P < 0.001$, by pairwise Wilcoxon rank sum test (B), and two-tailed Student's t test (D and F).

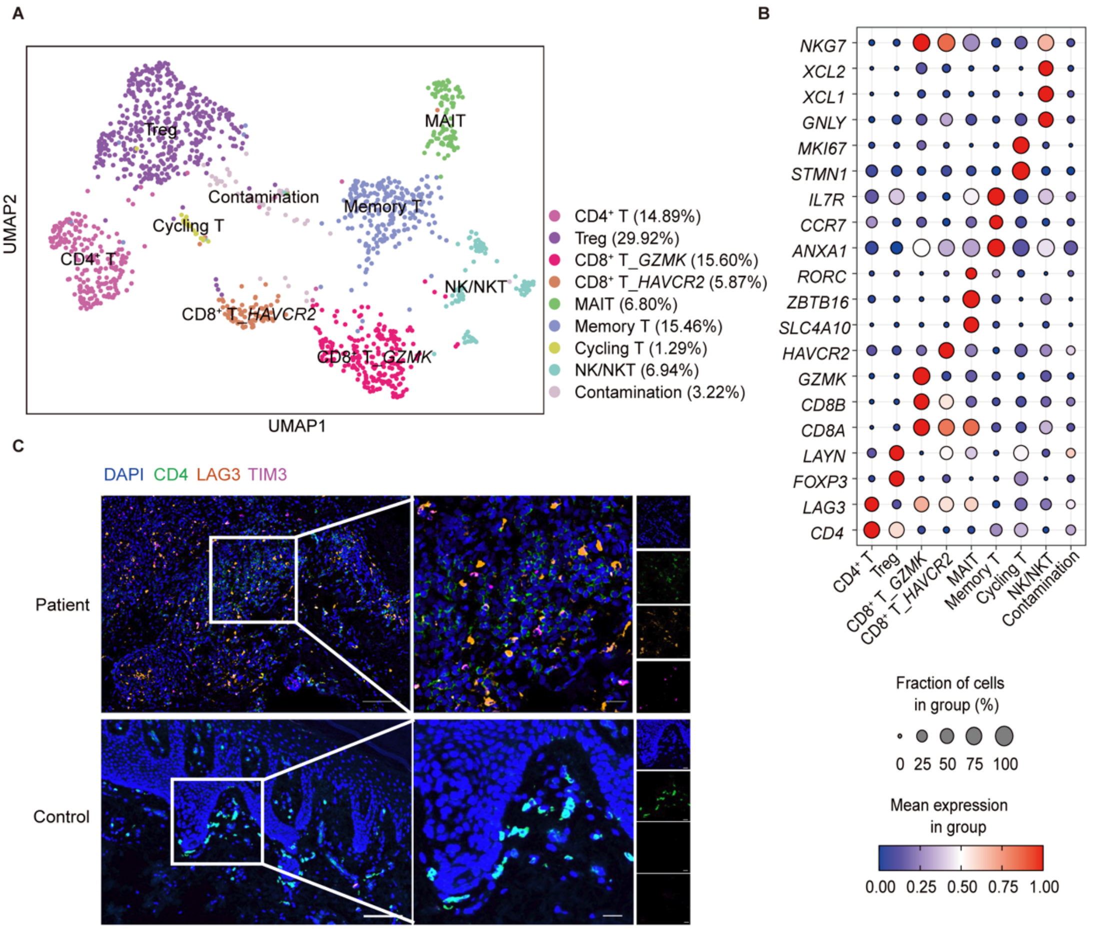

Supplementary Figure 3. Anti-inflammatory TREM2^{high} macrophages and exhausted T cells in lesions of CARD9-deficient patients with phaeohyphomycosis

(A) UMAP projection of T cells in the CARD9-deficient patient. Each point represented an individual cell, with the proportion of each subset within the total cell population annotated in the graph.

(B) Bubble chart shows mean relative expression of signature genes across the T cell subsets.

(C) Co-localization of CD4 and immune checkpoint (TIM3, and LAG3) in lesions of CARD9-deficient patients and controls. Scale bars, 100 μm (left) and 20 μm (right).

$^{***}P < 0.001$, by one-way ANOVA with Tukey's test (D).

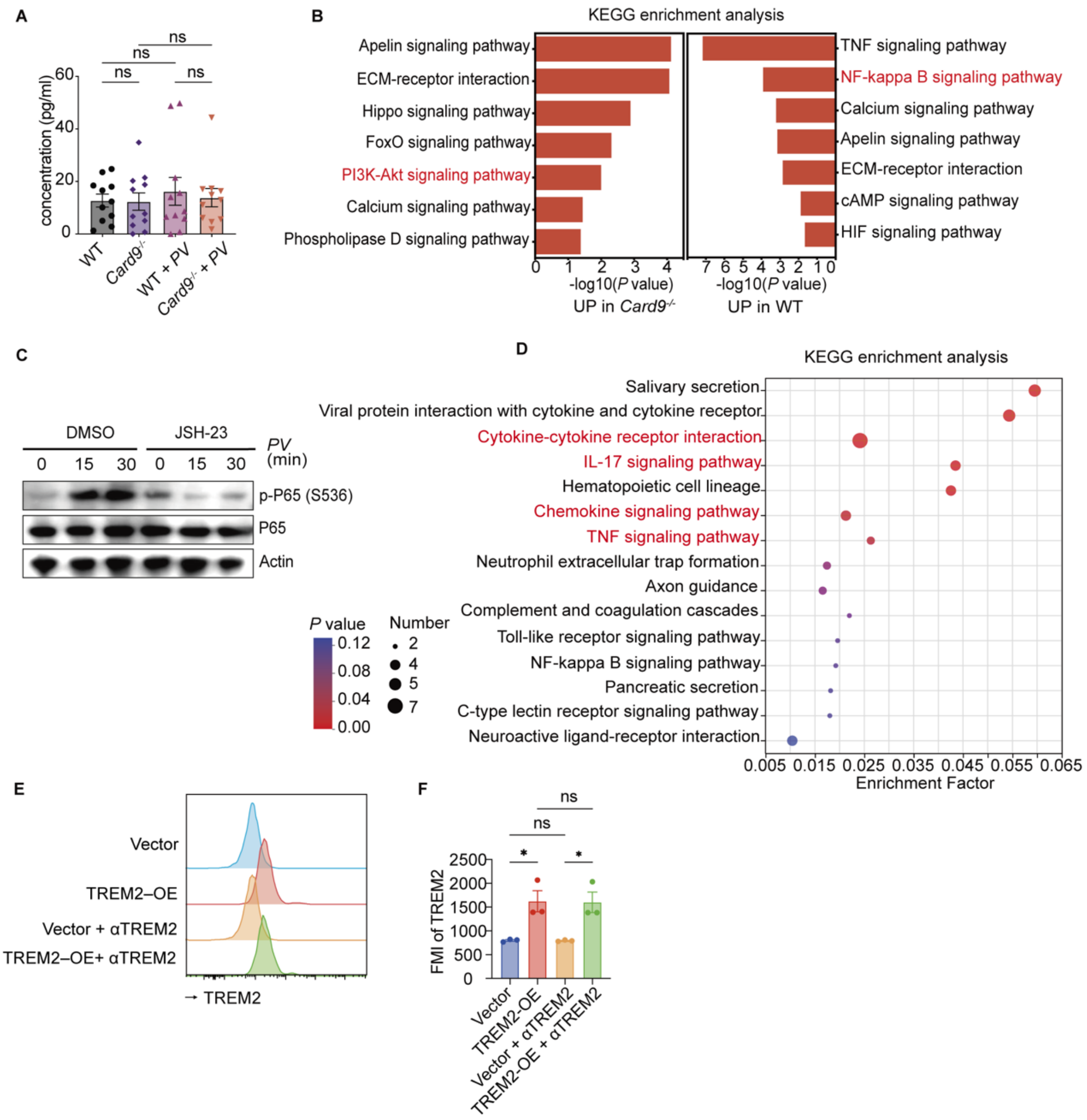

Supplementary Figure 4. CARD9 regulate TREM2 expression in macrophages and impairs antifungal infection

(A) Soluble TREM2 levels in the supernatant of WT and *Card9*$^{-/-}$ BMDMs after 24-hour stimulation with *P. verrucosa* (MOI = 10).

(B) BMDMs were stimulated with *P. verrucosa* (MOI = 10) for 24 h. Total RNA was extracted and RNA-Seq analysis was performed. KEGG pathway analysis of different genes of WT and *Card9*$^{-/-}$ BMDMs.

(C) Western blot analysis of total and phosphorylated p65 expression in WT BMDMs pretreated with DMSO or JSH-23, followed by stimulation with *P. verrucosa* (MOI = 10) for 15 or 30 minutes.

(D) Knockdown of endogenous TREM2 by RNA interference in WT and *Card9*$^{-/-}$ BMDMs using Lipofectamine 3000 transfection reagent. Cells were cultured for 48 h after transfection and then stimulated with heat-killed *P. verrucosa* conidia (MOI = 10) for 24 h. Total RNA was extracted and RNA-seq analysis was performed. The bubble plot shows KEGG enrichment analysis of upregulated genes in the si-*TREM2 Card9*$^{-/-}$ BMDM group.

(E) Histogram plots showing surface TREM2 expression in RAW264.7 cells transduced with control vector (blue) and TREM2-overexpressing (TREM2-OE) vector (red), as well as cells pretreated with anti-TREM2 antibody: control vector + anti-TREM2 (orange) and TREM2-OE + anti-TREM2 (green).

(F) Quantification of fluorescence mean intensity (FMI) of surface TREM2 expression from (E).

Data are shown as the mean ± SEM. ns $P > 0.05$, by one-way ANOVA with Tukey’s test (A and F).

Supplementary table 1. Clinical characteristics of CARD9-deficient patients with phaeohyphomycosis.

ID	Age (y)	Sex	Duration of infection (y)	Pathogenic fungus	Biopsy location	Therapy received at the time of biopsy	Co-existing illnesses	CARD9 mutations
P1	52	F	7	*Ochroconis musae*	Cheek	ITZ po. (400 mg/day) TBF po. (500 mg/day)	No	D274fsX60
P2	16	F	3	*Exophiala spinifera*	Cheek	ITZ po. (400 mg/day) TBF po. (500 mg/day)	No	S23X, D274fsX60
P3	29	M	5	*Phialophora verrucosa*	Left arm	ITZ po. (400 mg/ day)	No	R373P, D274fsX60

Supplementary table 2. Sequences of the target sequences siRNAs

siRNA	sequence (5′–3′)
h-si-*CARD9*	CCTCACGCATCACACCTTA
m-si-Trem2	AGAUGCUGGGCACCAACUUCATT
m-si-*Cebpbβ*-1	CGUUCGCCCUGCGCGCCUATT
m-si- *Cebpbβ*-2	AGCUGAGCGACGAGUACAATT
m-si- *Cebpbβ*-3	CACCCUGCGGAACUUGUUCAATT
si-NC	UUCUCCGAACGUGUCACGUTT

Supplementary table 3. Primer sequences for ChIP

	sequence (5′–3′)
Trem2 promoter-1	CTCTTTCTCCCTTTCTGCCTAC
Trem2 promoter-2	GCAAATGGGAACTCTGGCAC
Trem2 promoter-3	ATGACACCCCCCCTCAAATG

Supplementary Methods

Mice and cell lines

Wildtype C57BL/6J mice were purchased from Si Pei Fu Science (Beijing, China). *Card9*$^{-/-}$ mice (C57BL/6 background) were provided by Xin Lin (Tsinghua University School of Medicine, Beijing, China, and MD Anderson Cancer Center, Houston, TX). C57BL/6J and *Card9*$^{-/-}$ mice were maintained in specific pathogen-free animal facilities at Tsinghua University. In all the experiments described here, mice of the same sex and age were used. RAW 264.7 cell (TIB-71) and THP-1 cell (TIB-202) were purchased from American Type Culture Collection (ATCC).

BMDM preparation

Primary BMDM cultures from the indicated mice were prepared as previously described (2). Bone marrow cells were harvested from the femurs and tibias of the mice. The erythrocytes were lysed using ACK lysis buffer (Gibco). The cells were cultured for seven days in Dulbecco's modified Eagle medium containing 10% (v/v) fetal bovine serum, streptomycin (100 μg/mL), penicillin (100 U/mL), and 10% (v/v) conditioned medium from L929 cells expressing macrophage colony-stimulating factor.

THP-1 cell preparation

The human leukemia monocyte cell line THP-1 (ATCC) was cultured at 37 °C/5% (v/v) CO_2 in RPMI 1640 medium (Gibco) containing 10% (v/v) fetal bovine serum, streptomycin (100 μg/mL), and penicillin (100 U/mL). THP1 cells were differentiated into an adherent macrophage-like phenotype by treatment with 10 ng/mL phorbol 12-myristate 13-acetate (PMA, Sigma) for 48 h.

P. verrucosa stimuli preparation

The *P. verrucosa* strains were obtained from a CARD9-deficient patient and cultured on potato dextrose agar (BD Biosciences) for 14 days at 28 °C to harvest conidia. *P. verrucosa* was heated for 30 min at 99 °C in a water bath.

Flow cytometry

For flow cytometry analysis of macrophages, single-cell suspensions of murine lesions were stained with antibodies including FITC-conjugated anti-TREM2 (Invitrogen, MA5-28223), APC-conjugated anti-F4/80 (BioLegend, 123115), PcrCP-Cy5.5-conjugated anti-CD11b (BioLegend, 101227), PE-Cy7-conjugated anti-CD45 (BioLegend, 103113), and Zombie Aqua™ Fixable Viability (BioLegend, 423101) for 30 min at 4 °C in FACS buffer. For analysis of T cells, single-cell suspensions of murine lesion were stained with antibodies including FITC-conjugated anti-CD4 (BioLegend, 100405), APC-conjugated anti-CD3 (BioLegend, 100235), Brilliant Violet 421-conjugated anti-LAG3 (BioLegend, 125221), PE/Dazzle™ 594-conjugated anti-TIM3 (BioLegend, 134013), PE-Cy7-conjugated anti-CD45 (BioLegend, 103113), and Zombie Aqua™ Fixable Viability (BioLegend, 423101) for 30 min at 4 °C in FACS buffer. Finally, the stained cells were analyzed using a BD FACS flow cytometer, and the data were processed using Flowjo 10.4 software (TreeStar).

Histopathology

Skin lesions were fixed in 4% paraformaldehyde, processed routinely, embedded in paraffin, and sectioned at 5 μm. For hematoxylin and eosin (H&E) staining, sections were stained with hematoxylin, counterstained with eosin, dehydrated, cleared, and mounted with neutral resin. For Grocott's methenamine silver (GMS) staining, sections were oxidized in periodic acid solution, incubated in a preheated methenamine–silver working solution at 62 °C until black deposits appeared against a yellow–brown background, treated with sodium thiosulfate, counterstained with eosin, dehydrated, cleared in xylene, and mounted. All slides were examined and imaged using a light microscope (OLYMPUS, IX73).

Immunofluorescence staining

Formalin-fixed and paraffin-embedded tissues, sectioned to 4 μm, were utilized for the histologic evaluation of skin lesions in both human and mouse samples. Tissue slides were deparaffinized with xylene and rehydrated using a graded series of ethanol solutions (100%, 95%, and 70%). Subsequently, the slides were microwaved for 15 min to induce antigen retrieval using a citric acid solution. For mouse lesions, we utilized two panels of primary antibodies: (Panel 1) F4/80 (1:500, ab75677, Abcam) and TREM2 (1:200, ab213500, Abcam) and (Panel 2) CD4 (1:500, ab15580, Abcam), LAG3 (1:500, ab15580, Abcam), and TIM3 (1:500, ab15580, Abcam). For human samples, two panels of primary antibodies were used: (Panel 1) anti-mouse CD68 (1:500, ZM0060, ZSGB) and anti-rabbit TREM2 (1:50, ER1918-04, HUABIO) and (Panel 2) anti-rabbit CD4 (1:1000, ET1609-52, Abcam), anti-rabbit LAG3 (1:1000, 16616-1-AP, Proteintech), and anti-rabbit TIM3 (1:100, 45208S, CST).

Subsequently, the slides were incubated with secondary antibodies (1:1,100 μL for each slide; HRP-anti-rabbit IgG, ZSGB, PV-6001; or HRP-anti-mouse IgG, ZSGB, PV-6002) for 10 min at room temperature. Following each cycle of staining, heat-induced epitope retrieval was performed to

remove all primary and secondary antibodies. Multiplex immunofluorescence staining was performed using the AlphaTSA Multiplex IHC Kit (AXT36100031, AlphaX). The samples were counterstained with DAPI for 10 min and mounted in the mounting medium. Multispectral images were scanned using a ZEISS AXIOSCAN 7. The number of cells of interest was quantified using Halo (version 3.4; Indica Labs) and QuPath (version 0.2.0) software.

Single-cell RNA-sequencing analysis

Using CellRanger (v7.0.1), we aligned filtered FASTQ files with the reference genome to generate raw gene expression matrices (3). This process was applied using the GRCh38 (2020-A) and mm10 (2020-A) references for human and mouse samples, respectively. Raw gene expression matrices were imported for filtering using the Read10X function in Seurat (v4.0.3) (4). Briefly, genes expressed in fewer than three cells or cells with fewer than 300 genes were excluded. Additionally, cells exhibiting mitochondrial gene expression over 15% were also removed. The filtered data were then normalized using the NormalizeData function to adjust for variations in gene expression levels across cells, ensuring that differences in sequencing depth or cell size did not bias subsequent analyses. Subsequently, the FindVariableFeatures function was applied using the "vst" (variance-stabilizing transformation) method to identify 2000 highly variable genes. The ScaleData function was used to standardize the expression data of these genes. The RunPCA function was executed on the scaled data to reduce the dimensionality of the data and highlight the principal axes of variation. The FindNeighbors and FindClusters functions, which use PCA reduction, were used to identify groups of similar cells. Finally, to visually interpret these findings, RunUMAP was used to project high-dimensional data onto a two-dimensional space using the Uniform Manifold Approximation

and Projection (UMAP) method.

Removal of doublets

Doublets are artificial cells formed by co-encapsulating two or more cells during single-cell library preparation, which leads to inaccurate biological results. For this purpose, the DoubletFinder (v2.0.3) package was used, leveraging its algorithm to simulate artificial doublet formation and detect potential doublets within the dataset (5). Potential doublets were excluded from further analyses. In some cases, because the software was unable to find all doublets, we manually annotated the cells as doublets if they simultaneously expressed genes from two distinct cell lineages (e.g., marker genes for both T and B cells and marker genes for both myeloid and keratinocytes).

Integrating the datasets from all samples

To combine cellular information across different samples and mitigate batch effects, we employed a canonical correlation Analysis-based integration process. Initially, we identified the top 2,000 variable features that encapsulated the most significant aspects of cellular heterogeneity. The core integration process was then conducted using the FindIntegrationAnchors and IntegrateData functions. FindIntegrationAnchors identified common cell states across different datasets by finding 'anchors' between pairs of datasets. IntegrateData utilized these anchors to harmonize datasets and align them into a single integrated dataset.

Developmental trajectory analysis

Diffusion mapping is a technique used for dimensional reduction and is particularly useful in single-

cell RNA sequencing (scRNA-seq) data analysis to uncover the underlying structure of the data, such as developmental trajectories or cell differentiation pathways. In our study, we applied the diffusion map method from the R package destiny (v3.4.0) to elucidate the developmental stages of T cell differentiation from naïve to various effector and exhaustion states (6). This process involved calculating the distances between T cells based on their expression profiles, constructing an affinity matrix, and computing a diffusion map to identify the principal components of variation. The diffusion components derived from the eigendecomposition provided a reduced-dimensional representation of the T cell data, highlighting the continuous nature of T cell differentiation.

Visualization and statistics

The single-cell RNA sequencing data were predominantly visualized using a suite of R packages, such as Seurat and ggplot2 (v3.4.2). Additional support for specific visual tasks was provided by scRNAtoolVis (v0.0.4) for bubble plots showing marker gene expression and by scatter (v1.22.0) and irGSEA (v2.1.5) for specialized plotting functions tailored to single-cell data and gene set enrichment analyses, respectively. Other figures in our manuscript were generated using the default visualization functions available within the R packages detailed in the Methods section. We meticulously annotated the statistical details of each figure in the captions to ensure clarity and transparency of the analyses.

RNA sequencing and analysis

Total RNA was extracted from the cells using TRIzol® Reagent (Diagen), according to the manufacturer’s instructions. The RNA-seq transcriptome library was prepared following Illumina®

Stranded mRNA Prep, Ligation (San Diego, CA) using 1μg of total RNA. Libraries were size selected for cDNA target fragments of 300 bp on 2% LowRange Ultra Agarose, followed by PCR amplification using Phusion DNA polymerase (NEB) for 15 PCR cycles. After quantification using Qubit 4.0, sequencing was performed on a NovaSeq X Plus platform (PE150) using the NovaSeq Reagent Kit. The raw paired-end reads were trimmed, and quality was controlled using fastp with default parameters. High-quality reads were aligned with those of Homo sapiens (https://www.ncbi.nlm.nih.gov/genome/). To identify differential expression genes (DEGs) in different samples, the expression level of each transcript was calculated according to the transcription-per-million reads method. DEGs with $|\log2FC| \geq 1$ and $FDR < 0.05$ were considered to be significantly different expressed genes. GO functional enrichment and KEGG pathway analyses were performed using Goatools and Python SciPy software, respectively.

Western blot assays

BMDM and differentiated-THP1 cells were treated with heat-killed *P. verrucosa* conidia (MOI=10) for 15 min to 24 h and lysed in lysis buffer (immunoprecipitation assay buffer containing protease and phosphatase inhibitors). The cell lysates were subjected to immunoblotting with the indicated antibodies. Primary antibodies used were anti-phospho-P65 Ser536 (3033T, CST), anti-total P65(ab32536, Abcam), anti-phospho-AKT S473 (4060T, CST), anti-total AKT (4691T, CST), anti-phospho-GSK3β Ser9 (558T, CST), anti-total GSK3β (9315T, CST), anti-phospho-CREB S133 (ab32096, Abcam), anti-total CREB (ab32515, Abcam), anti-phospho-C/EBPβ Thr235 (3084T, CST), anti-total C/EBPβ (ab32358, Abcam), anti-TREM2 (ab209814, Abcam), anti-TREM2 (ab305103, Abcam), anti-β-actin (66009-1-Ig, Proteintech), and anti-GAPDH (60004-1-Ig,

Proteintech). The secondary antibodies used were horseradish peroxidase-linked anti-rabbit (7074S, CST) and anti-mouse (7077S, CST) antibodies.

RNA interference

Interfering RNAs targeting human CARD9, mouse Cebpb, mouse Trem2, and non-silenced controls were designed and synthesized by Shanghai GenePharma Company (Shanghai, China). Lipofectamine 3000 transfection reagent (Thermo Fisher Scientific) was used to transfect siRNA according to the manufacturer's protocol. The target sequences are listed in Supplementary Table 1.

Dual-luciferase reporter assay

Luciferase assays were performed as previously described (7). In brief, HEK293T cells were transfected with a set of plasmids comprising a reporter construct derived from pGL3 containing the Trem2 promoter region inserted 5′-upstream of firefly luciferase, along with a C/EBPβ expression plasmid (PCDNA3.1-Mouse_Cebpb), and a control reporter plasmid encoding EF1α promoter-driven Renilla luciferase. The transfected cells were cultured in DMEM containing 10% FBS for 48 h, collected, and lysed. Firefly and Renilla luciferase activities were measured using the Dual-Luciferase Reporter System, and Renilla luciferase activity was used to normalize transfection efficiency.

Chromatin immunoprecipitation assays

The Chromatin immunoprecipitation (ChIP) assay was performed using a ChIP kit (53009, Active Motif) as previously described (8). The antibodies used for ChIP were anti-C/EBPβ (sc-7962, Santa

Cruz Biotechnology). The resulting DNA was analyzed by real-time PCR. The PCR primers used are listed in Supplementary Table 2.

In vitro fungal killing assay

In vitro fungal killing assay was performed as described previously (2). Briefly, BMDMs were mixed with live *P. verrucosa* conidia (MOI = 10) for 30 min at 37 °C. After removing unbound particles, the cells were incubated for 30 or 120 min. At the indicated time points, the medium was removed, and cells were lysed with 1% Triton X-100. Finally, the lysis buffer was diluted and plated on Sabouraud's agar for 10 days, and the CFU was determined.

Soluble TREM2 ELISA

BMDMs were stimulated with heat-killed *P. verrucosa* for 24 hours. Supernatants were collected, and soluble TREM2 levels were measured using an ELISA kit (JL20435–96T, JONLNBIO) according to the manufacturer's instructions.

Reference

1. Mulder K, et al. Cross-tissue single-cell landscape of human monocytes and macrophages in health and disease. Immunity. 2021;54(8):1883-900 e5.
2. Zhao X, et al. JNK1 negatively controls antifungal innate immunity by suppressing CD23 expression. Nat Med. 2017;23(3):337-46.
3. Zheng GX, et al. Massively parallel digital transcriptional profiling of single cells. Nat Commun. 2017;8:14049.
4. Butler A, et al. Integrating single-cell transcriptomic data across different conditions, technologies, and species. Nat Biotechnol. 2018;36(5):411-20.
5. McGinnis CS, et al. DoubletFinder: Doublet Detection in Single-Cell RNA Sequencing Data Using Artificial Nearest Neighbors. Cell Syst. 2019;8(4):329-37 e4.
6. Haghverdi L, et al. Diffusion pseudotime robustly reconstructs lineage branching. Nat Methods. 2016;13(10):845-8.
7. Xu X, et al. CARD9S12N facilitates the production of IL-5 by alveolar macrophages for the induction of type 2 immune responses. Nature Immunology. 2018;19(6):547-+.
8. Zhao XQ, et al. C-type lectin receptor dectin-3 mediates trehalose 6,6'-dimycolate (TDM)-induced Mincle expression through CARD9/Bcl10/MALT1-dependent nuclear factor (NF)-kappaB activation. J Biol Chem. 2014;289(43):30052-62.
