# Supplementary material for: CARD9-dependent macrophage plasticity regulates effective fungal clearance
Source: J Clin Invest. 2025 Dec 2;136(3):e188827. doi: 10.1172/JCI188827 (PMC12867132; doi:10.1172/JCI188827)
Supplement: Unedited blot and gel images [file jci-136-188827-s297.pdf]

Figure 5B

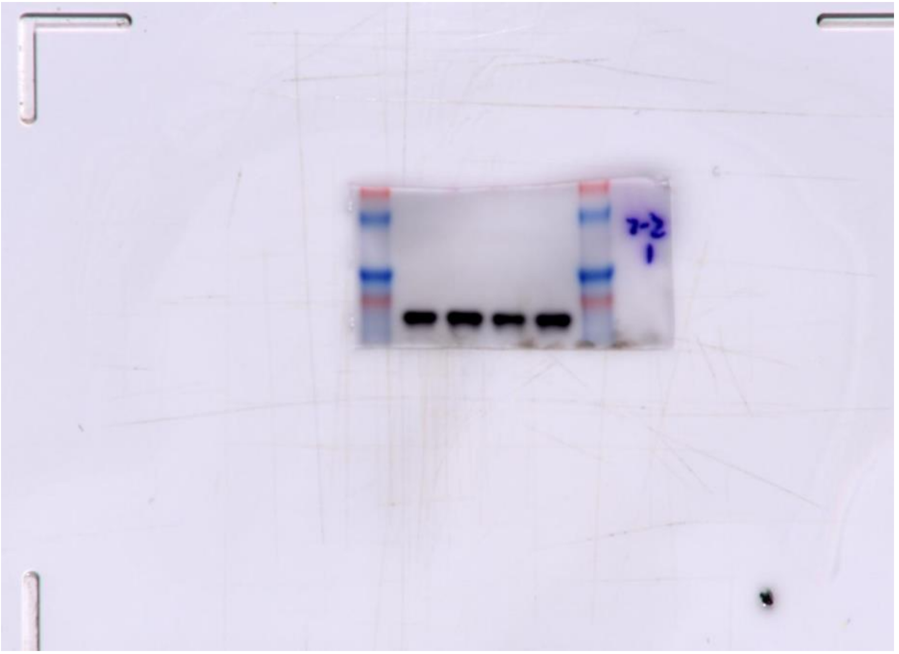

Actin

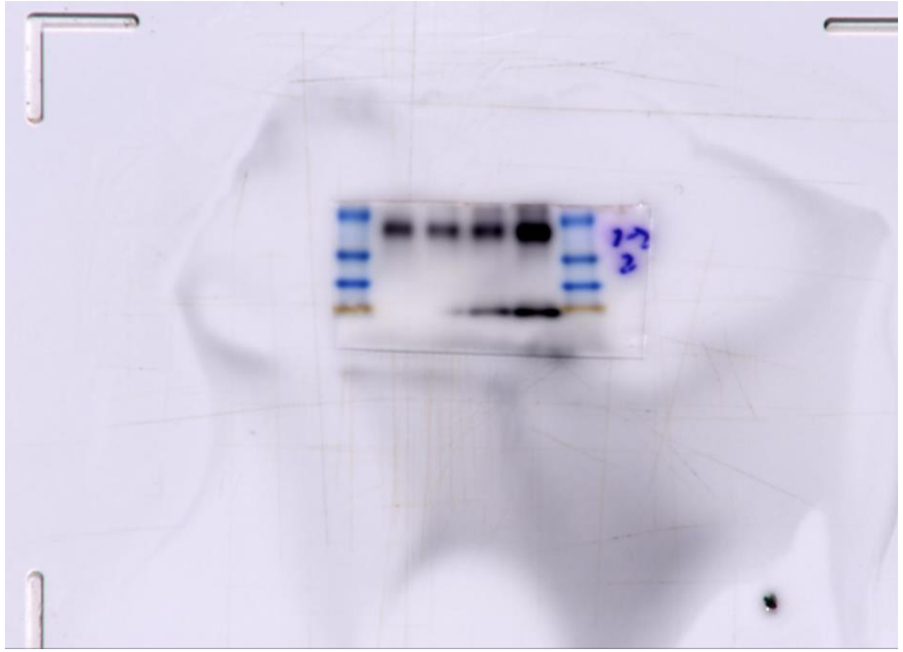

TREM2

Figure 5F

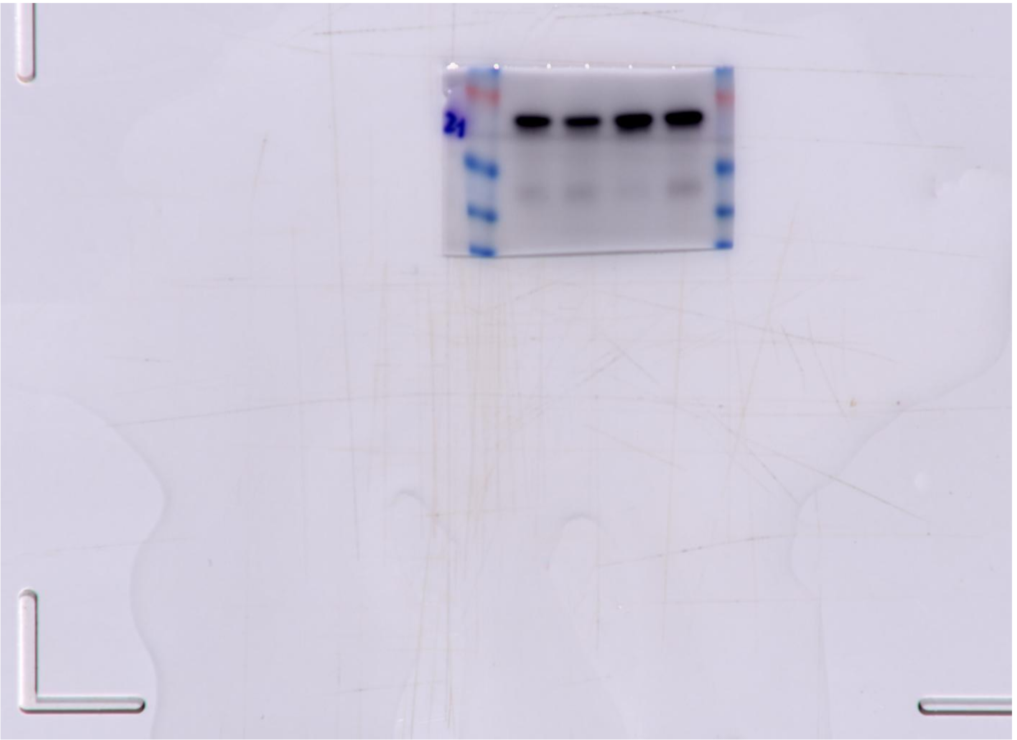

Actin

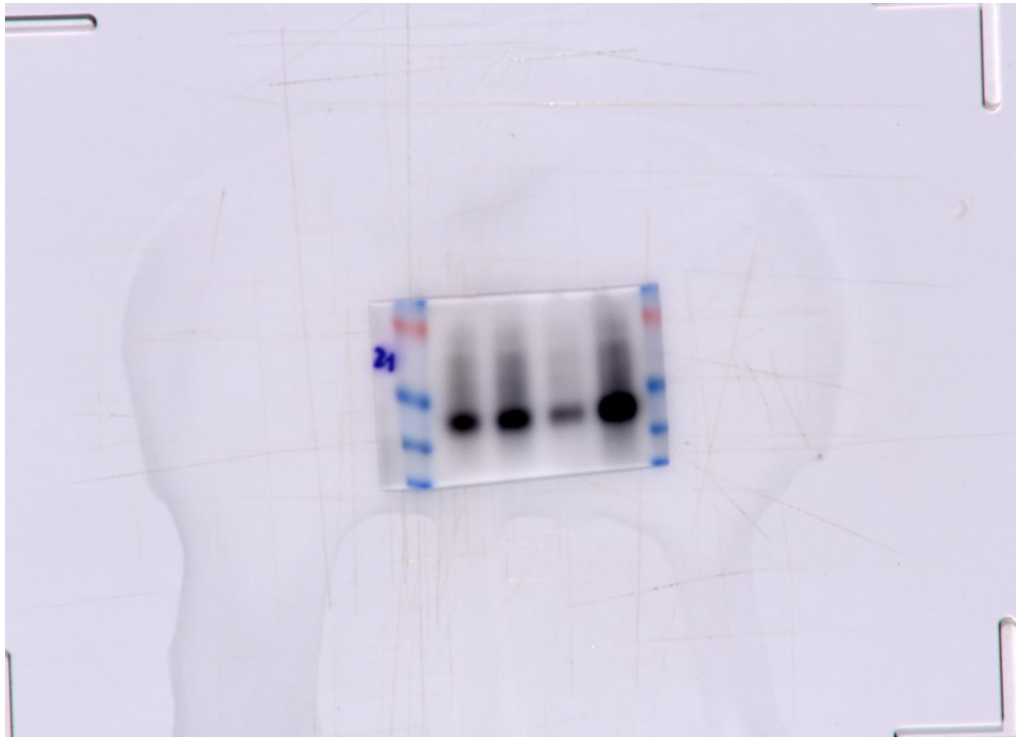

TREM2

Figure 5G

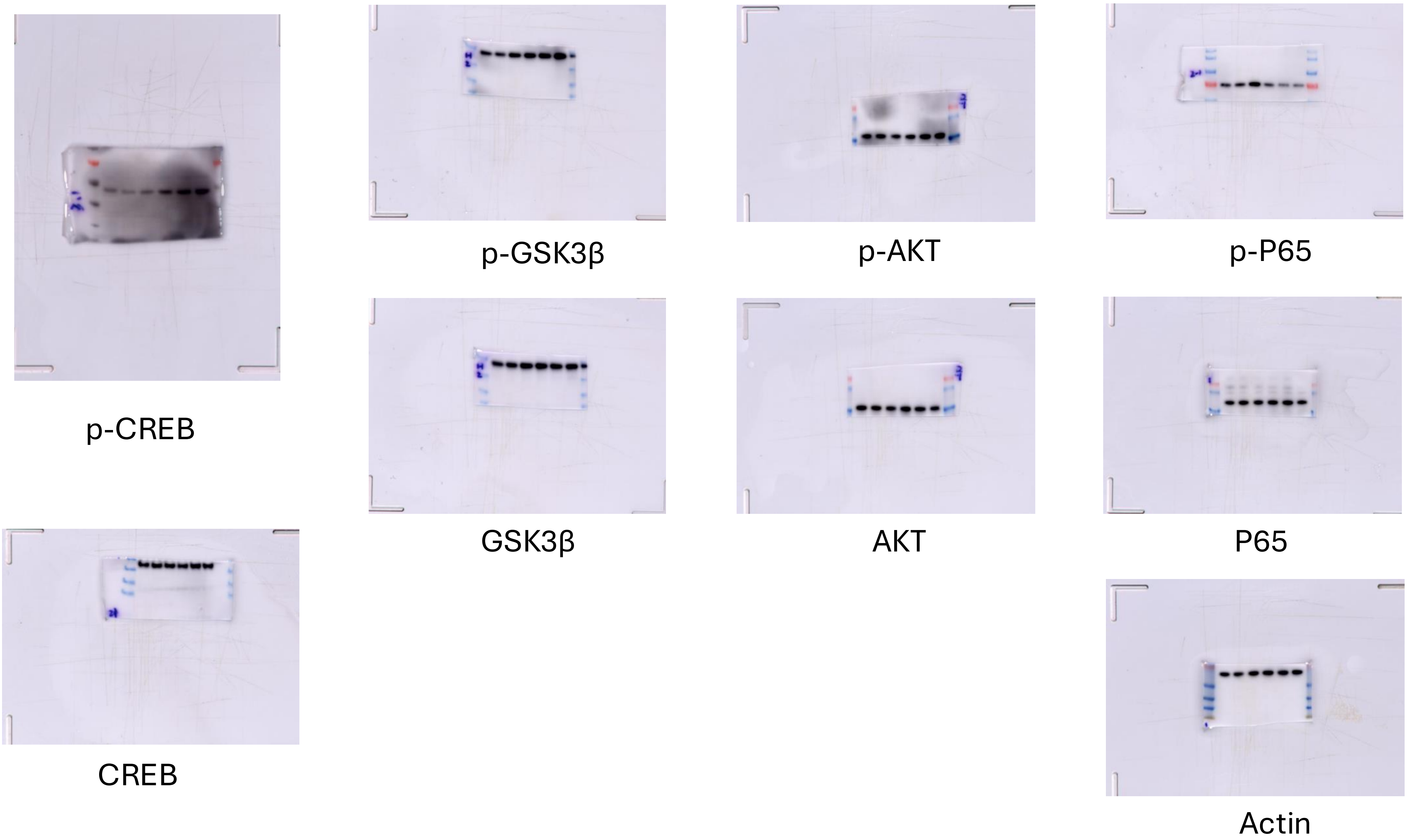

Figure 5I

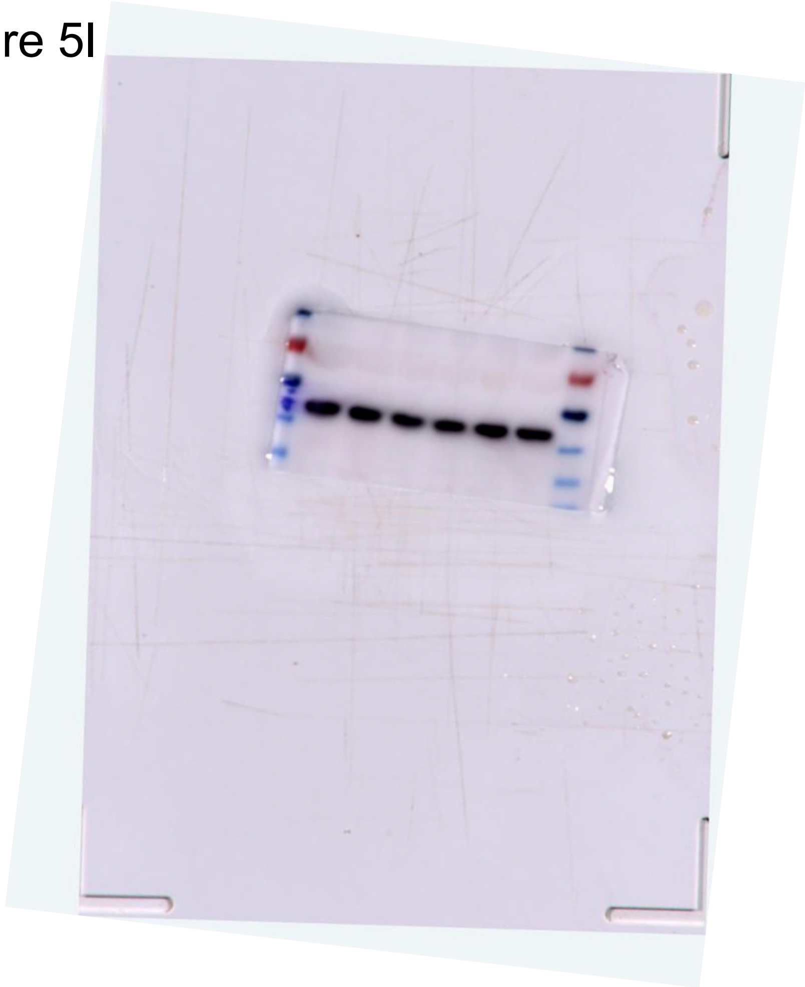

Actin

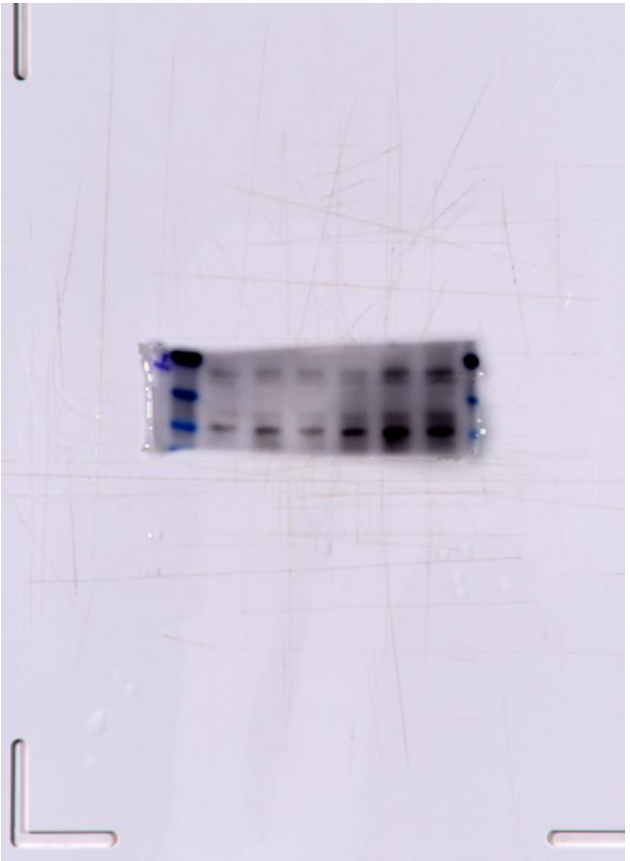

p-CREB

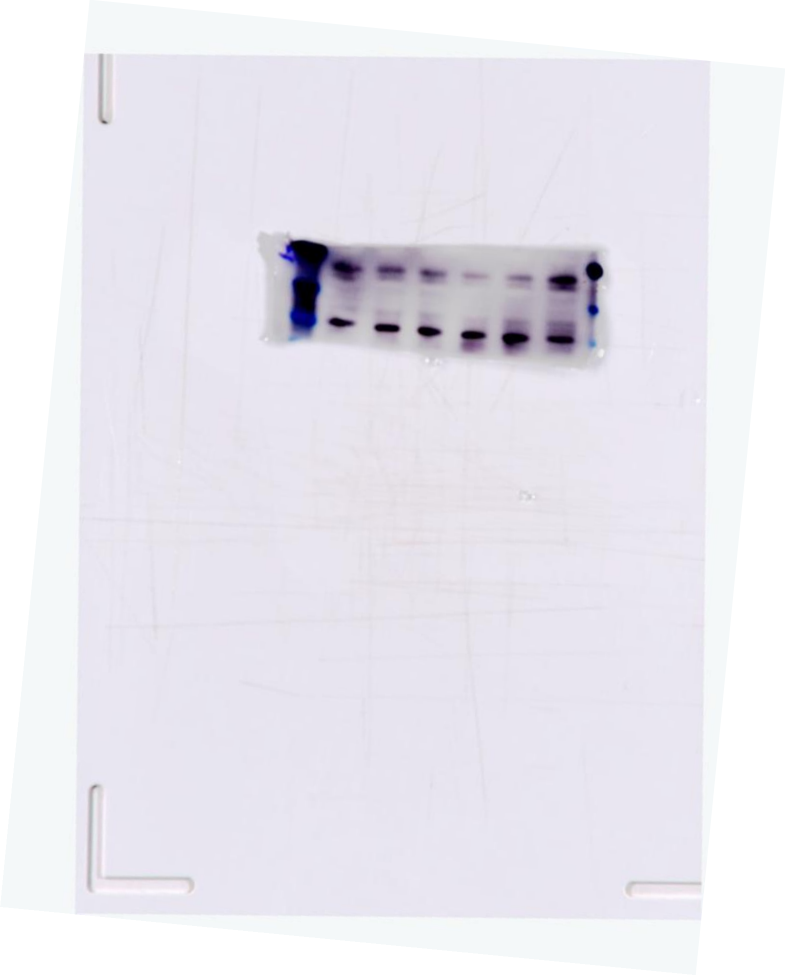

CREB

Figure 5J

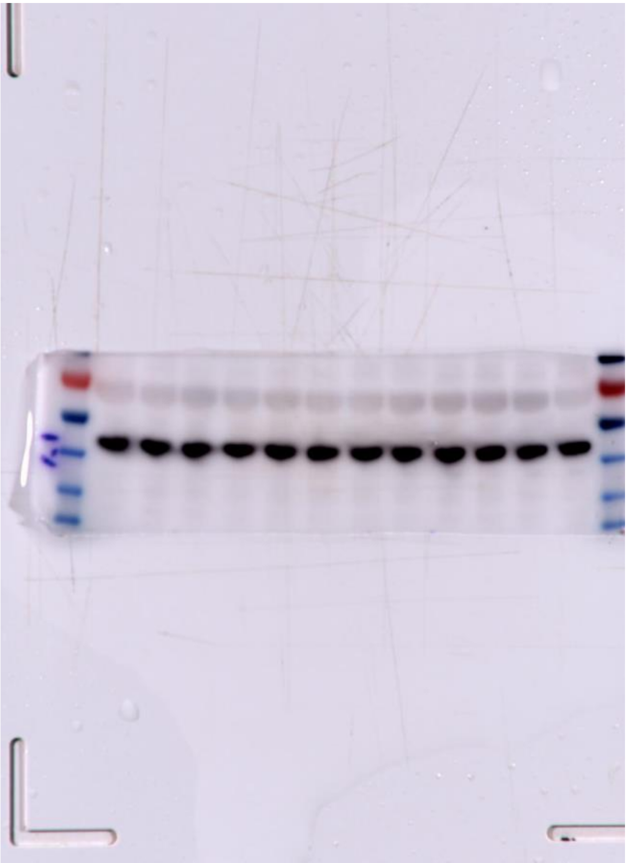

Actin

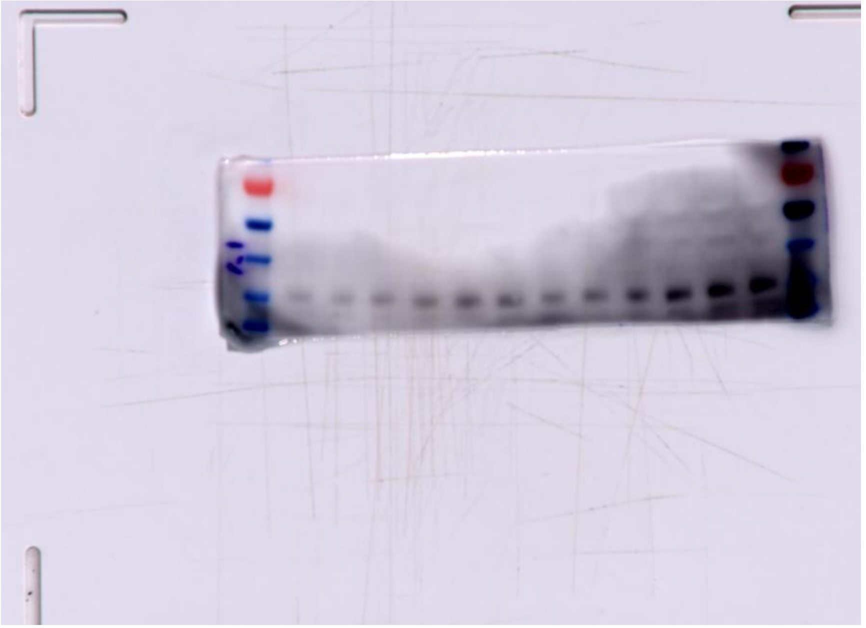

TREM2

Figure 5R

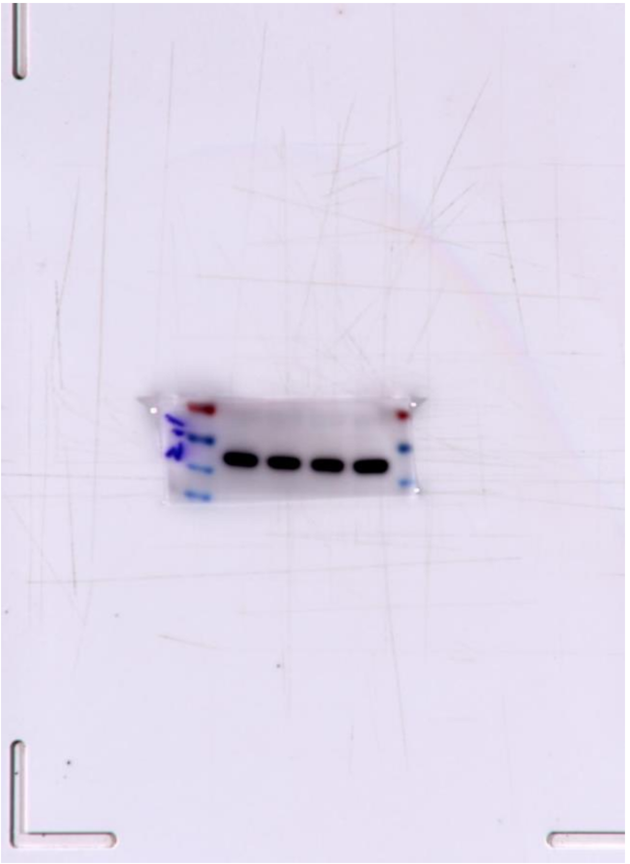

Actin

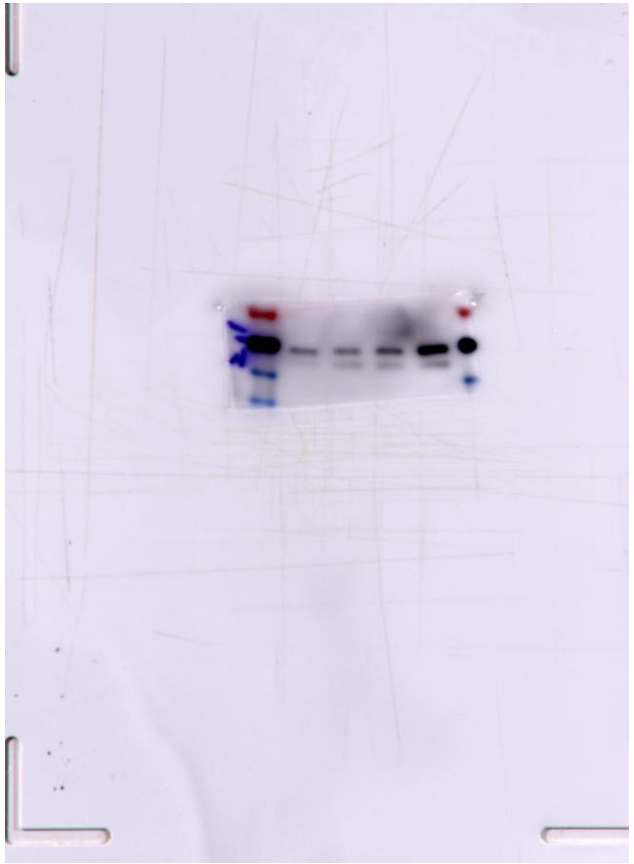

TGF-β

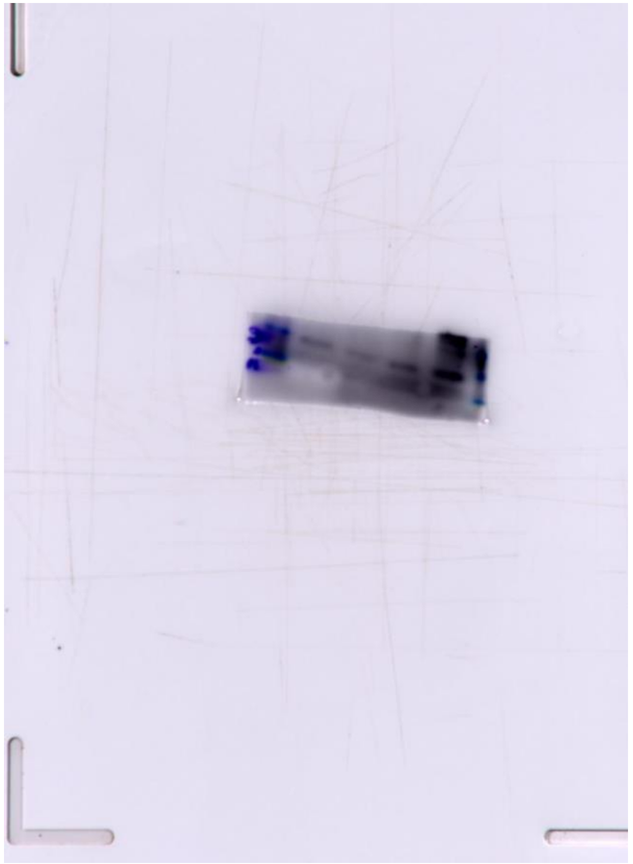

IL-10

Figure 6B

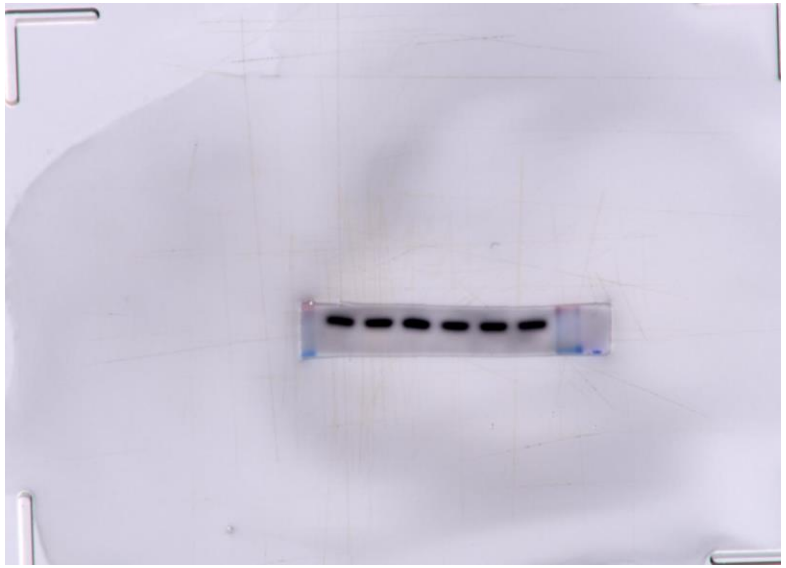

Actin

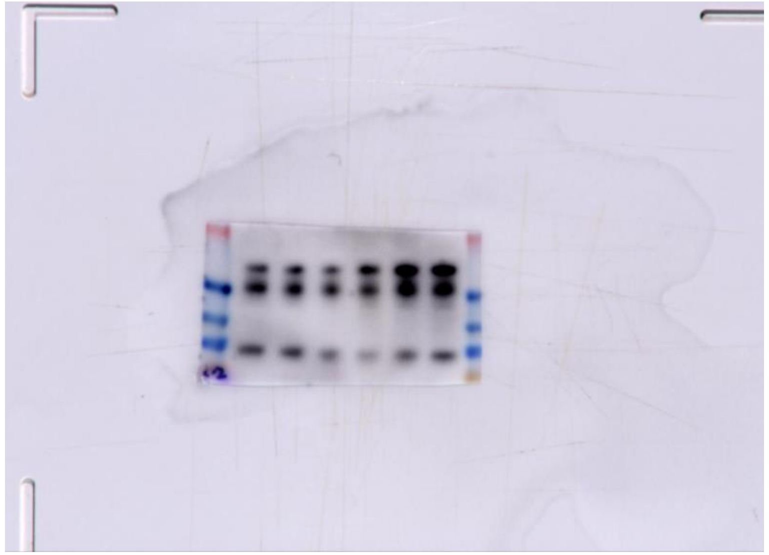

p-C/EBPβ

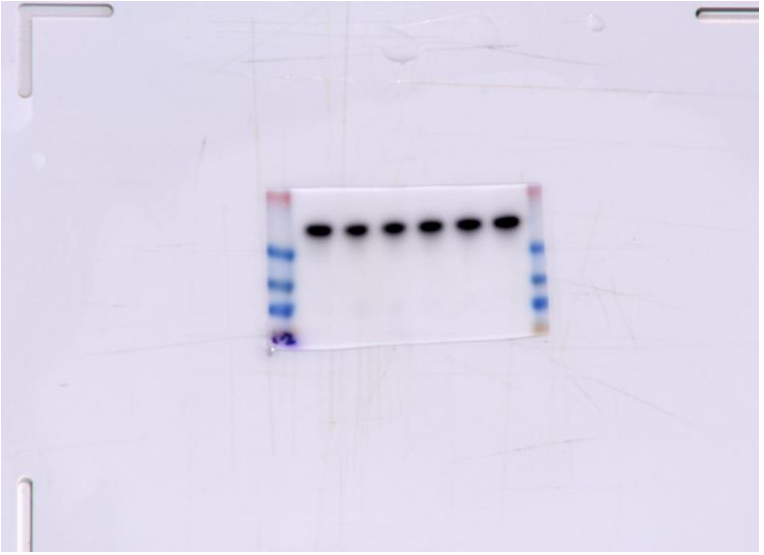

C/EBPβ

Figure 6D

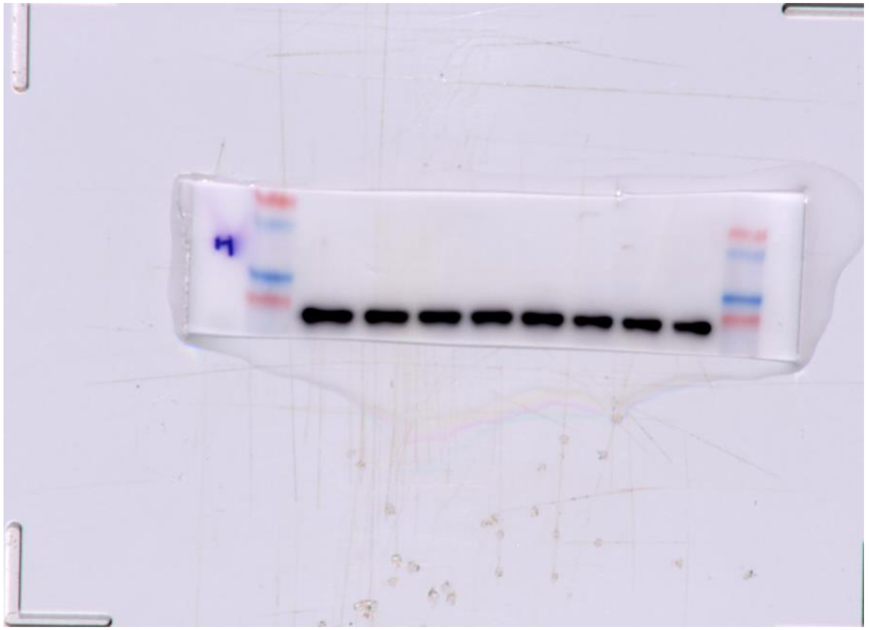

Actin

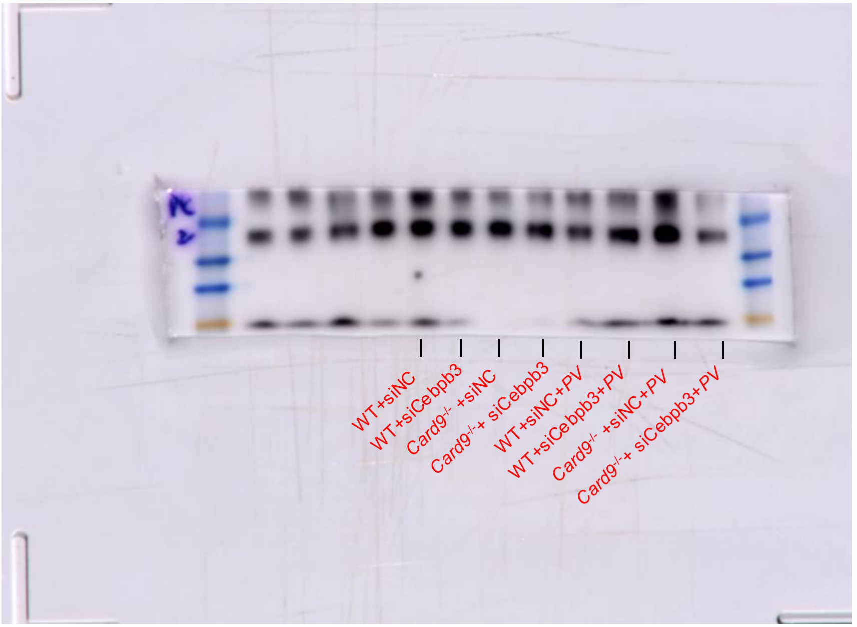

TREM2

Figure S4C

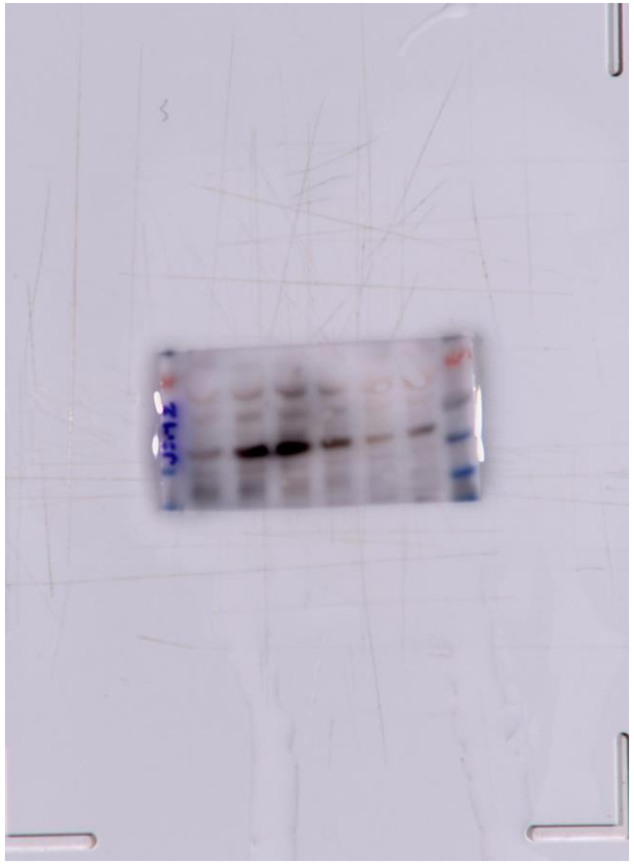

p-P65

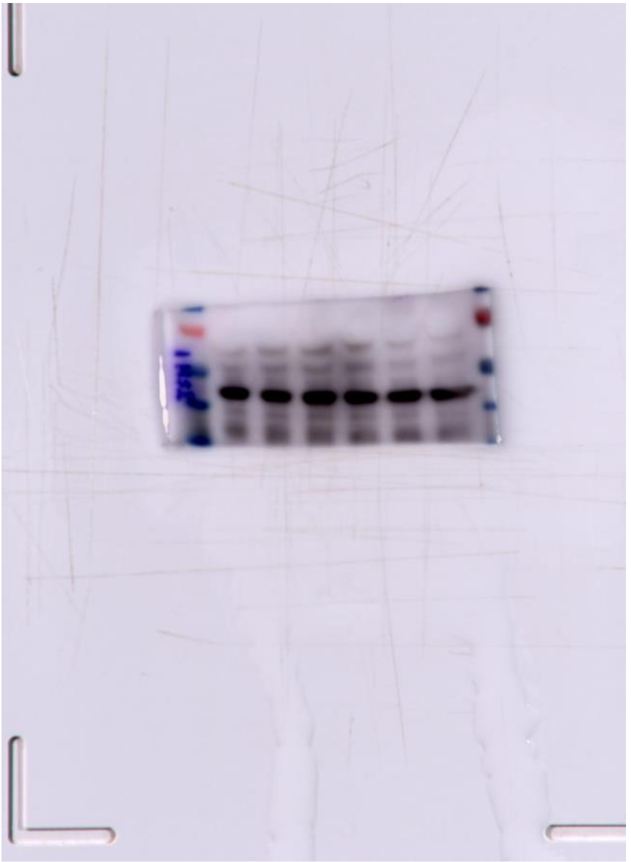

P65

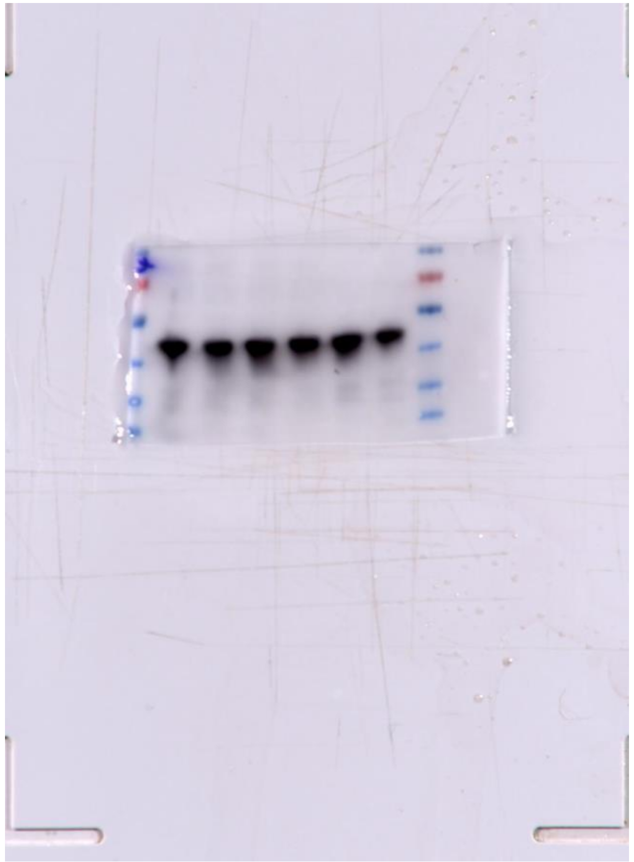

Actin
